# Trypsin-instructed bioactive peptide nanodrugs with cascading transformations to improve chemotherapy against colon cancer

**DOI:** 10.1186/s12951-025-03143-1

**Published:** 2025-01-31

**Authors:** Can Wu, Xiao Wei Zhang, Manman Wang, Jinpan Sun, Jianfei Chen, Yanbin Guan, Xin Pang

**Affiliations:** 1 School of Pharmacy, Henan University of Chinese Medicine, Zhengzhou, 450046 China; 2 Collaborative Innovation Center of Research and Development on the Whole Industry Chain of Yu-Yao, Henan University of Chinese Medicine, Zhengzhou, Henan Province 450046 China; 3 Academy of Chinese Medicine Science, Henan University of Chinese Medicine, Zhengzhou, 450046 China

**Keywords:** Bioactive peptide, Cascading transformations, Chemotherapy, Trypsin, Colon cancer

## Abstract

Chemotherapy remains an effective treatment for colon cancer but is hampered by its limited response rate. Bioactive peptides, marked with intracellular transformations, have been reported as an effective approach to boosting chemotherapeutic activity. Herein, a promising trypsin-responsive bioactive peptide-based nanodrug is constructed, which could significantly prolong the drug retention time in vivo by cascading transformations and improve chemotherapeutic efficacy. Initially, 1-Pept co-assembles with Dox into a few nanofibers called 1-Pept/Dox NFs, inducing an enhanced cellular uptake *via* caveolae-mediated endocytosis by avoiding lysosomal degradation and further promoting perinuclear transportation, thus enlarging the drug efficacy in target areas. After nanofiber disassembly, the released 1-Pept converts into Pept under the catalysis of intracellular overexpressed trypsin, which then reassembles into denser Pept NFs, inducing a cascade of effects including disruption of the cytoskeleton, mitochondrial dysfunction, and activation of caspase-3. By the synergism of Pept NFs and Dox, caspase-3 can be further activated, and cause greater damage to nuclear, thereby leading to tumor ablation. As the first example of employing trypsin-mediated nanodrugs with cascading transformations to promote chemotherapeutic activity, this work promises a strategy for novel therapies for efficiently combating colon cancer.

**Graphical Abstract:**

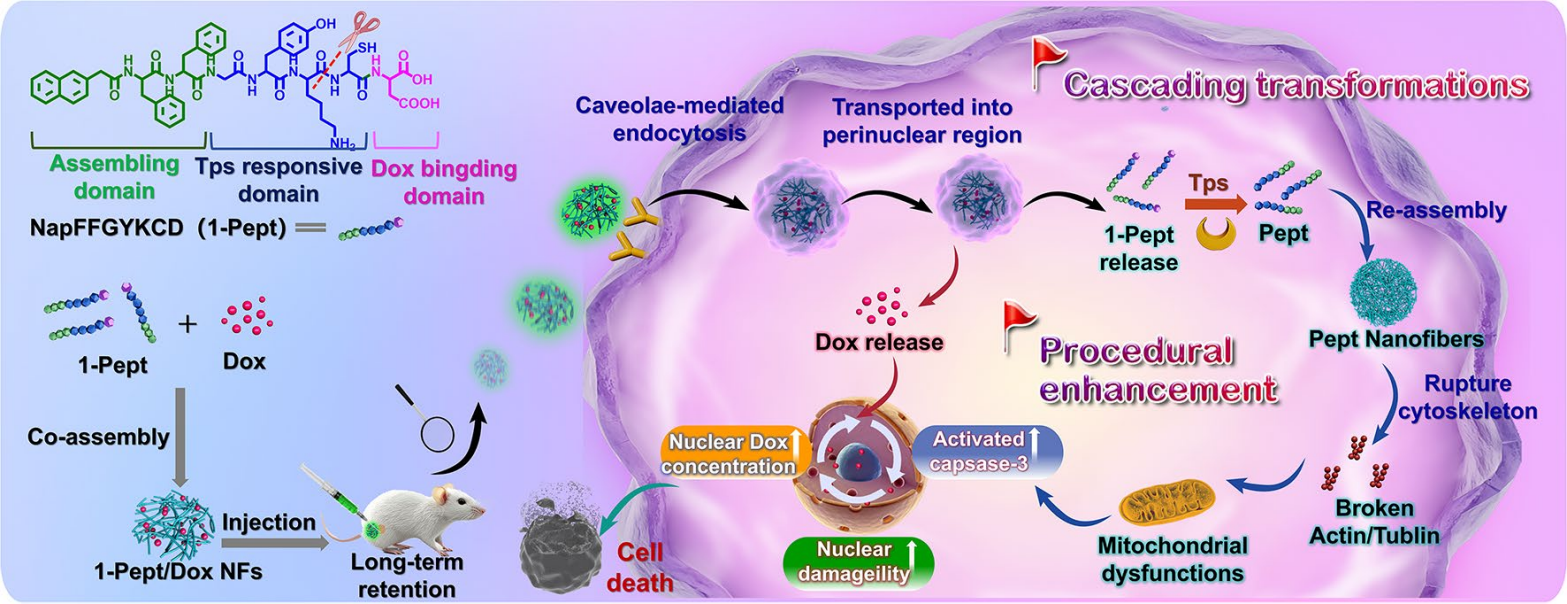

**Supplementary Information:**

The online version contains supplementary material available at 10.1186/s12951-025-03143-1.

## Introduction

Colon cancer is the world’s fourth cause of cancer, with nearly 900,000 deaths annually [1–3]. According to the genetics, epigenetics and clinical features of colon cancer, three subtypes are identified as CCS1, CCS2 and CCS3. Different subtypes show different disease-free survival and diverse responses to therapeutic drugs [4]. Among them, CCS3 tumors show important heterogeneity in phenotypic and genetic characteristics, with strong aggressive behavior and poor prognosis. As one of the conventional treatment methods for colon cancer, chemotherapy is demonstrated to be effective in reducing disease burden. However, commonly used chemotherapeutic agents, including 5-fluorouracil, 10-hydroxycamptothecin, and cisplatin, frequently bring adverse effects such as infections, allergic reactions, ototoxicity, and nephrotoxicity [5, 6]. In comparison, doxorubicin (Dox) has a broader anti-cancer spectrum, and it can avoid toxic side effects such as phlebitis caused by long-term and continuous infusion of drugs like 5-fluorouracil. Besides, it also exhibits good therapeutic effects on numerous tumor types, notably breast cancer, lung cancer, and bladder cancer. It has been proved that Dox is an effective adjuvant chemotherapy drug for advanced colon cancer, exerting its anticancer effects through binding to DNA and inhibiting the synthesis of nucleic acids [7]. However, the clinical deployment of Dox has many challenges. High-dose administration may induce cardiotoxicity [8]. It exhibits nonselective distribution in vivo, resulting in low drug concentration in target areas and short retention in the body [9]. To address the challenges above, a more efficient delivery system should be developed to expand Dox’s clinical application. Peptide-based self-assembly drug delivery system offers several distinct advantages for the treatment of colon cancer, including excellent biocompatibility, controlled and sustained drug release profile, and targeted drug delivery ability to the lesion sites, promising a strong potency in chemotherapy [10–12]. However, there are several challenges when using self-assembled peptides as drug carriers, including limited drug loading capacity as being introduced in large quantities, the unsatisfactory in vivo stability of peptides-based carriers, and low yields accompanied by complex synthesis when two or more drugs are introduced the same time, which to some extent limit the further application of peptide-based delivery systems [13, 14].

A growing number of studies have shown that specific sequence peptides possess multiple roles: they can not only assemble into multifunctional nanocarriers for drug delivery but also exert bioactivity effects, including anti-cancer, anti-bacterial, and anti-inflammatory [15–17]. Impressed by their favorable functions, bioactive peptides are marked with the following superiorities in cancer therapy: (1) Enhanced cellular uptake of drugs by multiple drug-loaded forms, including covalent conjugation, physical encapsulation, or co-assembly with drugs [18–20]; (2) Prolonged drug retention time in vivo [21]; (3) Improved anti-cancer effects by cooperating with therapeutic agents [22, 23]. More importantly, the special microenvironments of the lesion sites in the body, such as acidic pH, overexpressed enzymes, and high concentrations of GSH [24, 25], provide favorable conditions to regulate the assembly process of active peptides spatiotemporally. This could cause disorder of cell membrane structure, destruction of cytoskeleton structure, damage of nuclear function, and activation of body immunity, thus exerting stronger therapeutic effects [26–29].

Among them, enzyme-instructed peptide self-assembly (EISA) can regulate the in-situ self-assembly or re-assembly of peptides around and inside cells and at the site of the subcellular organelles. This is due to the overexpression of enzymes in the tumor microenvironment, which induces a series of impacts. Such damage can occur to cell membrane structure, cytoskeleton structure, or subcellular organelles; therefore, research studies employing this strategy have demonstrated significant advances and enhanced cell death selection [30–33]. By applying the overexpressed enzyme in a tumor, domestic and foreign research groups have developed multiple EISA-based drug delivery systems. These systems will not only control in-situ morphological transition but also realize the selective killing of cancer cells [34–36]. Although EISA has exhibited advantages in cancer therapy, such as high selectivity and long retention in vivo, it still can be improved in the efficiency of cancer treatment [37, 38]. Recent studies indicate that an EISA-based delivery system will not only enhance the retention time in the body by cascading transformations but also boost the activity of chemotherapy drugs, offering a new strategy for colon cancer treatment [39, 40]. Trypsin (Tps), a digestive enzyme produced by pancreatic acinar cells, plays a crucial role in protein digestion. Abnormal Tps levels can be indications of various medical conditions, including cancer, acute pancreatitis, and inflammation [41, 42]. Increasing data suggest that overexpressed Tps in colon cancer is closely associated with colon cancer progression [43]. Soreide et al. demonstrated in a study that overexpressed Tps was distributed in the cytoplasm of colon cancer cells, which may be crucial for colon cancer metastasis [44]. Thus, a new strategy of manipulating bioactive peptide in-situ assembly by highly expressed Tps in colon cancer sites may be an efficient method for combating colon cancer.

**Scheme 1:**
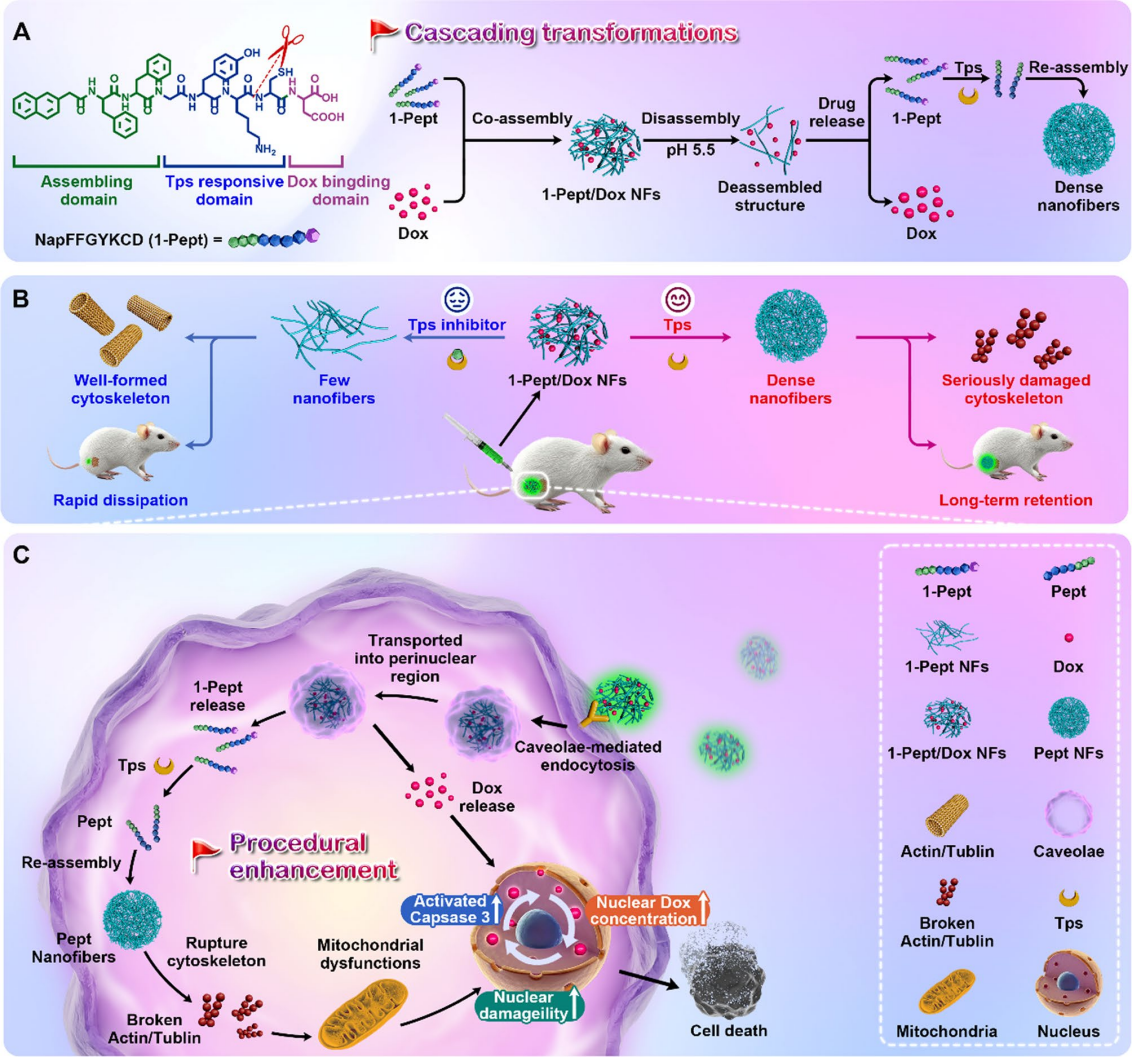
Schematic illustrations of Tps-instructed bioactive peptide nanodrugs with cascading transformations to promote chemotherapy against colon cancer. (**A**) Chemical structure of Tps responsive 1-Pept and the cascading transformations of co-assembled 1-Pept/Dox NFs. (**B**) 1-Pept/Dox NFs responded to Tps into dense nanofibers for disruption of the cytoskeleton and long-term retention in vivo. (**C**) 1-Pept/Dox NFs with cascading transformations to better promote chemotherapy for efficiently killing colon cancer cells

In this study, trypsin-instructed peptide self-assembly-based nanodrugs are developed, possessing cascading transformations for a promoted chemotherapeutic activity against colon cancer. As shown in Scheme 1, a self-assembling bioactive peptide precursor (NapFFGYKCD, termed 1-Pept) is prepared, which consists of (i) Naphthylacetic acid-Phenylalanine-Phenylalanine (NapFF), a well-studied sequence for promoting assembly; (ii) a Tps-responsive peptide sequence Glycine-Tyrosine-Lysine-Cysteine (GYKC), which could cleave at the carboxyl side of lysine (K) [45, 46]; and (iii) Aspartic acid (D) at the C-terminus for binding with Dox. 1-Pept is co-assembled with Dox into nanofibrillar hydrogels (denoted as 1-Pept/Dox NFs) *via* non-covalent interaction, inducing a cascade of deformations under an acidic environment and overexpressed Tps in colon cancer (Scheme 1A), thus leading to serious damage to the cytoskeleton of colon cancer cells with long-term retention in vivo (Scheme 1B). Upon exposure to colon cancer sites, 1-Pept/Dox NFs exhibit an enhanced cellular uptake *via* caveolae-mediated endocytosis, which can avoid lysosomal degradation, further promote perinuclear transportation, enlarge the chemotherapeutic drug potency in the target areas, and thus augment the anti-cancer efficacy. Subsequently, 1-Pept and Dox are released from 1-Pept/Dox NFs upon reaching the acidic perinuclear region. Dox could enter the nucleus and intercalate into DNA, further activating caspase-3 and causing nuclear damage. The released 1-Pept converts into NapFFGYK (termed Pept) upon the intracellular overexpressed Tps catalysis, which reassembles into denser Pept nanofibers (denoted as Pept NFs). After that, Pept NFs induce a cascade of effects, including disruption of the cytoskeleton, mitochondrial dysfunction, and activation of caspase-3. By the synergism of Pept NFs and Dox, caspase-3 is further activated and cause greater damage to cancer cell nucleus, thereby achieving improved treatment efficacy (Scheme 1C). This work, the first example of employing the overexpressed Tps-instructed nanodrugs with cascading transformations, provides a new strategy for optimizing chemotherapeutics medication against colon cancer.

## Results and discussion

### Trypsin-instructed molecular assembly of 1-Pept for triggering intracellular cascade reactions

An increasing number of studies have shown that Tps plays an important role in the proliferation and invasion of colon cancer cells [47, 48]. To better explore the effect of overexpressed Tps on the treatment of colon cancer, we designed and synthesized a Tps responsive bioactive peptide that produced some cell mechanisms to improve the efficacy of the treatment against colon cancer. The sequence of the designed peptide is 2-Naphthylacetic acid-Phe-Phe-Gly-Tyr-Lys-Cys-Asp, which includes the following parts: (I) 2-Naphthylacetic acid-Phe-Phe served as a common hydrophobic sequence for facilitating the assembly process; (II) Gly-Tyr-Lys-Cys (GYKC), designed as a Tps-responsive sequence; (III) The amino acid Asp (D) was chosen to adjust the hydrophilic-hydrophobic equilibrium of the peptide (Scheme S1A). It could also co-assemble with Dox into nanostructure *via* electrostatic interaction. The structure of the synthesized peptide was purified by the RP-HPLC method and verified by mass spectrometry (MS) and ^1^H-NMR (Fig. S1-S2).

**Fig. 1:**
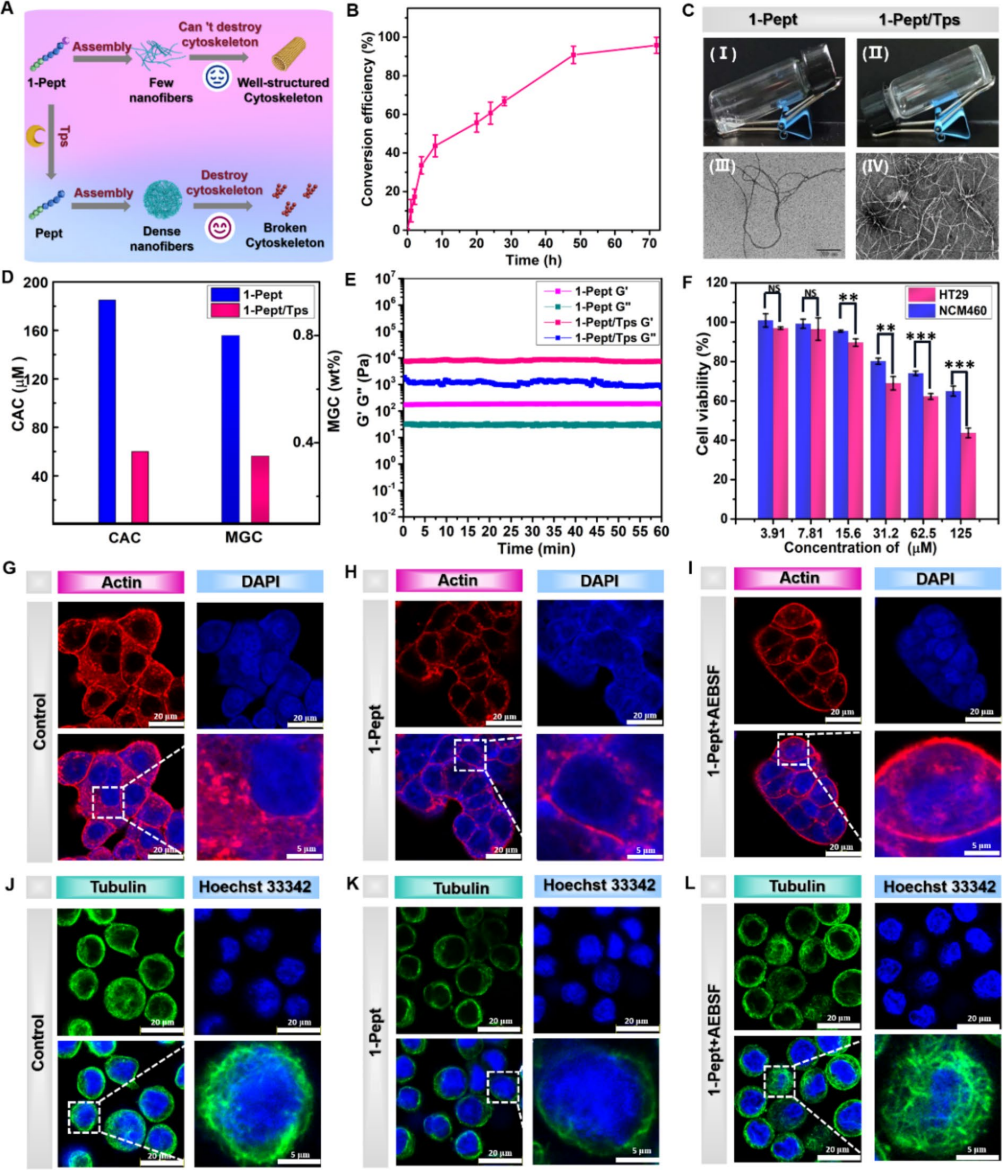
Characterizations of Tps-instructed molecular assembly of 1-Pept. (**A**) Schematic diagram of the morphological transformation of 1-Pept catalyzed by Tps, which could transform from few nanofibers to dense nanofibers and destroy the cytoskeleton. (**B**) Conversion efficiency of 1-Pept (1 mg/mL) after treatment of Tps (1.0 U/mL). (**C**) Gelation performance of 1-Pept (0.5 wt%) before (I) and after (II) Tps treatments, and transmission electron microscope (TEM) images of 1-Pept (1.0 wt%) before (III) and after (IV) Tps treatments. Bar, 200 nm. (**D**) Values of critical aggregation concentration (CAC) and minimum gelation concentration (MGC) of 1-Pept NFs and 1-Pept/Tps NFs. (**E**) Dynamic time scanning of 1-Pept NFs and 1-Pept/Tps NFs. (**F**) Cell viabilities of HT29 and NCM460 cells after 48 h treatment of 1-Pept. (**G**) CLSM images of HT29 cells to indicate intracellular actin in the control group. (**H**) CLSM images of disruption of intracellular actin after HT29 cells receiving 1-Pept treatment for 12 h. (**I**) CLSM images of intracellular actin after HT29 cells received 2 h pretreatment of Tps inhibitor (AEBSF) and further 1-Pept treatment for 12 h. Red, Alexa Fluor 633 phalloidin; Blue, DAPI; Bar, 20 μm; Bar in magnified images, 5 μm. (**J**) CLSM images of HT29 cells for indicating intracellular tubulin in the control group. (**K**) CLSM images of intracellular tubulin in HT29 cells after receiving 1-Pept treatment for 12 h. (**L**) CLSM images of intracellular tubulin in HT29 cells after receiving pretreatment of AEBSF for 2 h and further 1-Pept treatment for 12 h. Green, Tubulin Tracker™ Green; Blue, Hoechst 33,342-indicated nucleus. Bar, 20 μm. Bar in magnified images, 5 μm

The structure of NapFFGYKCD contains Tps-responsive sequence GYKC, which would convert into NapFFGYK (Scheme S1B) under the intracellular overexpressed Tps of colon cancer cells (Fig. 1A). The enzyme catalytic kinetics experiment was first investigated by treating 1-Pept solution with Tps for HPLC-MS analysis. As shown in Fig. S3-S4, the new peak of Pept was observed after treatment of Tps in 1-Pept solution and Pept peak area increased with time. Within 72 h, more than 90% of 1-Pept converted into Pept after treatment of Tps (Fig. 1B). Subsequently, we evaluated the role of Tps in regulating the assembly process of 1-Pept. 1-Pept (0.5 wt%) formed a transparent solution in the absence of Tps in PBS solution (pH = 7.4) (Fig. 1C (I)), which would convert into stable fibrillar hydrogel 1-Pept/Tps NFs under the catalysis of Tps (5 U/mL) (Fig. 1C (II)). Few nanofibers in 1-Pept NFs (1.0 wt%) were observed by transmission electron microscopy (TEM) measurement, which was attributed to the weak assembly ability (Fig. 1C (III)). Encouragingly, denser nanofiber was observed in 1-Pept/Tps NFs (1.0 wt%), which proved the indispensable role of Tps in boosting the assembly process (Fig. 1C (IV)). We also measured the critical aggregation concentration (CAC) and minimum gelation concentration (MGC) of 1-Pept and 1-Pept/Tps NFs (Fig. 1D). The value of CAC for 1-Pept/Tps NFs was 60 µM, which was lower than that of 1-Pept NFs (185 µM), and the value of MGC of 1-Pept/Tps NFs (0.35 wt%) was significantly lower than that of 1-Pept NFs (0.8 wt%). Further circular dichroism (CD) test indicated that 1-Pept and 1-Pept/Tps NFs exhibited a positive peak near 195 nm and a negative peak at 216 nm, confirming a β-sheet conformation in the fibrillar hydrogels (Fig. S5).

The viscoelasticity of 1-Pept and 1-Pept/Tps NFs was evaluated by a rotational rheometer. As shown in Fig. S6, dynamic strain sweep indicated that with the increase of the applied strain, the storage modulus values (G’) and loss moduli (G’’) of 1-Pept NFs were decreased, suggesting an obvious shear thinning performance. When the critical shear stress values reached 45%, the gelation-solution transition was formed. Dynamic time sweep demonstrated that the value of G’ of 1-Pept/Tps NFs was 47 times higher than that of 1-Pept NFs under equal conditions, proving the reinforcement effects of Tps on the assembly of 1-Pept/Tps NFs (Fig. 1E). These viscoelastic properties of the 1-Pept NFs are conducive to the development of injectable preparation for delivery of chemotherapeutic agents.

Mounting evidence suggested that enzymatic intracellular transformations could lead to cell death by destroying the cytoskeleton structure [49]. Since the Tps expression level in colon cancer cells was higher than in normal colon cells, we first determined the intracellular Tps activity using a trypsin activity quantitative determination kit. As shown in Fig. S7, the intracellular Tps expression of human colon cancer cells (HT29) reached 0.3 mU/mL, which equaled 3.2 fold, 5.4 fold, and 1.5 fold of NCM460 cells, A375, and HeLa cells, respectively, showing clearly the high level of Tps in HT29 cells. Thereafter, we investigated the cytotoxicity of 1-Pept towards HT29 cells and human normal colon epithelial cells (NCM460). Surprisingly, the cell survival rates of 1-Pept-treated HT29 cells were lower than that of 1-Pept-treated NCM460 cells within the measured concentration range, which could be attributed to the overexpressed Tps-mediated intracellular assembly in HT29 cells (Fig. 1F). HT29 or NCM460 cell lysate was obtained and added into a 1-Pept solution to investigate the intracellular Tps-mediated assembly. As demonstrated in Fig. S8, the treatment of the 1-Pept solution with HT29 cell lysate resulted in gelation. Conversely, incubation with blank culture or NCM460 cell lysate maintained the solution state, suggesting that Tps may play a role in facilitating the assembly process of 1-Pept. For further confirming Tps-induced intracellular assembly, HT29 cells were treated with 1-Pept for TEM observation. The results depicted in Fig. S9 indicate that a dense nanofiber network was observed in the cellular lysates of HT29 cells incubated with 1-Pept. In contrast, only a few nanofibers were present in the cellular lysates of HT29 cells pre-incubated with the Tps inhibitor (AEBSF). This finding provides evidence that Tps plays a role in facilitating the intracellular assembly process of 1-Pept.

Further investigation was needed to elucidate the mechanism by which 1-Pept-mediated EISA induced cytotoxicity in colon cancer cells. The morphological changes of the cytoskeleton after HT29 cells receiving 1-Pept treatments were measured. As shown in Fig. 1G, in the control group, the bright red fluorescence signals of Alexa Fluor 633 phalloidin-staining actin were observed under confocal laser scanning microscopy (CLSM). In contrast, faint fluorescence signals were detected after 1-Pept treatments (Fig. 1H). We also observed that the control group exhibited well-defined, stretched-thin actin filaments. The 1-Pept-treated group showed disrupted actin arrangements, and most of the actin was shrunk in the cell membrane. Notably, actin filaments were recovered in the Tps inhibitor AEBSF-pretreated group (Fig. 1I). To understand further the role of the EISA process on the cytoskeleton, we also investigated the changes in microtubules using Tubulin Tracker™. As shown in Fig. 1J, K and a significant decrease in green fluorescence signals of Tubulin Tracker^TM^-staining tubulin and distinct changes in microtubule structure were also observed after the treatment with 1-Pept. Furthermore, the AEBSF-pretreated group exhibited tiny changes in microtubules (Fig. 1L). These findings suggested that the disruption of cytoskeleton structure was a plausible mechanism of 1-Pept-induced cytotoxicity. Previous studies have confirmed that damage to the cytoskeleton can trigger a decrease in mitochondrial function, inducing cell apoptosis and ultimately leading to nuclear damage. Considering this, we evaluated the 1-Pept-induced mitochondrial dysfunction. As shown in Fig. S10, control cells exhibited bright red fluorescent signals of JC-10 aggregation and faint green fluorescent signals of JC-10 monomer. In contrast, 1-Pept-incubated HT29 cells showed decreased red fluorescence signals but enhanced green fluorescence signals, indicating the decreased cell mitochondria membrane potential (ΔΨ_m_). Pre-incubation of AEBSF with HT29 cells resulted in an increase in red fluorescence signal intensity and a decrease in green fluorescence signal intensity compared with the 1-Pept group, suggesting that inhibition of intracellular Tps activity could reverse the decrease in ΔΨ_m_ induced by 1-Pept. Further study indicated that mitochondrial dysfunction activated caspase-3, for the green fluorescence signals of GreenNuc™ caspase-3 substrates were significantly enhanced compared with the control cells (Fig. S11). Following the pre-incubation of HT29 cells with AEBSF, there was an attenuation in the intensity of intracellular green fluorescent signal, indicating a reduction in the activation of apoptotic proteins induced by 1-Pept. Subsequently, we examined DNA damage by measuring the expression level of H2A.X, a biomarker for indicating DNA damage. Notably, the green fluorescence signal intensity of 1-Pept-treated cells was distinctly stronger than that of the control group, indicating that 1-Pept induced noticeable DNA lesions. Furthermore, the study demonstrated that the DNA damage caused by 1-Pept could be reversed in the presence of Tps inhibitors, as shown in Fig. S12. Overall, the EISA triggered by 1-Pept could initiate a series of intracellular reactions, such as disruption of the cytoskeleton, dysfunction of mitochondria, and damage to the nucleus, ultimately resulting in the death of cancer cells.

#### 1-Pept co-assembled with Dox into 1-Pept/Dox NFs with enhanced assembly capability

**Fig. 2:**
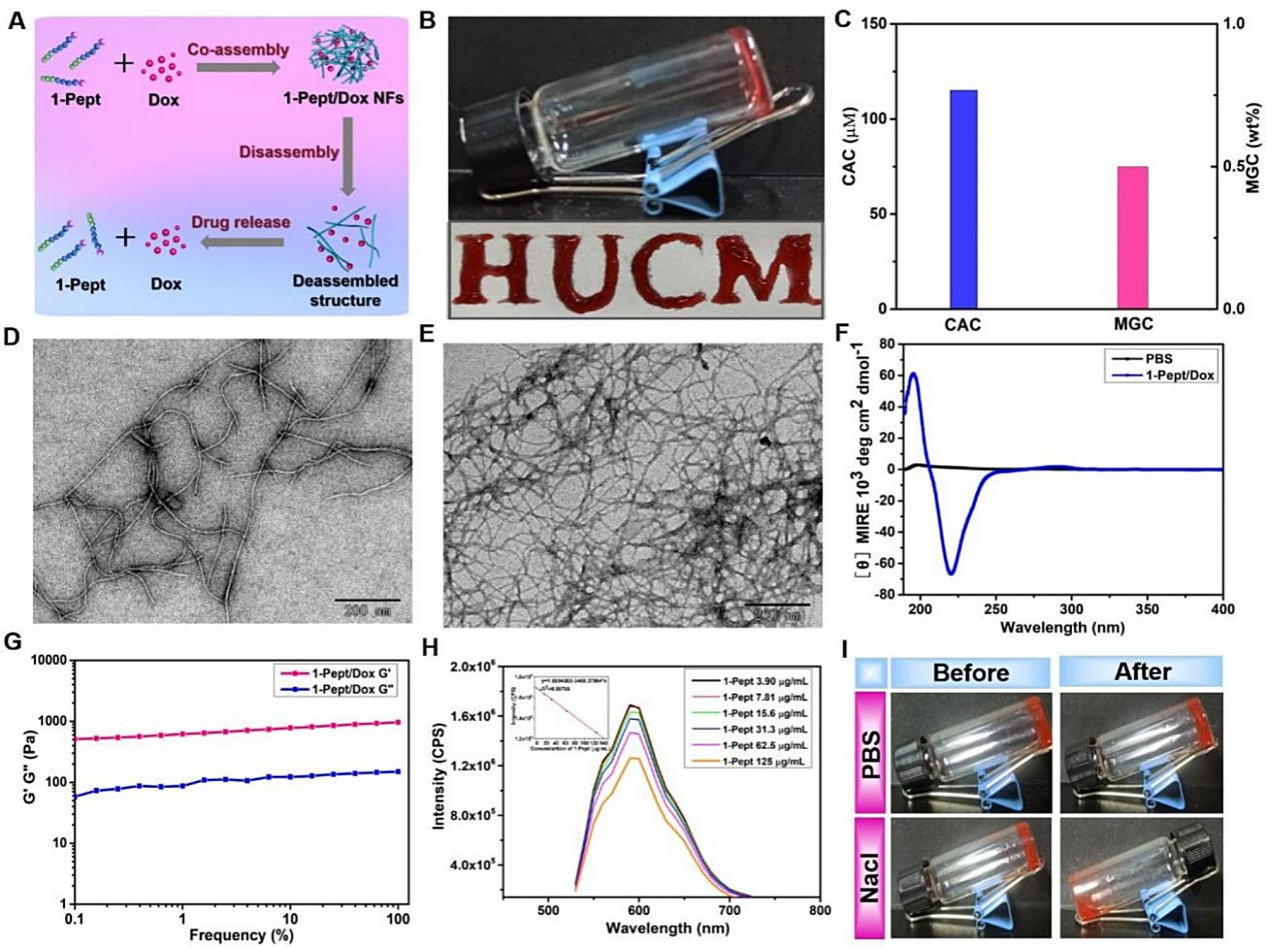
Characterizations of 1-Pept/Dox NFs with enhanced assembly capability. (**A**) Schematic diagram of 1-Pept co-assembled with Dox into 1-Pept/Dox NFs. (**B**) Top: Optical images of 1-Pept/Dox NFs (1.0 wt%/0.1 eq); Bottom: Optical images of the “HUCM” letter written by 1-Pept/Dox NFs with a syringe needle. (**C**) CAC and MGC values of 1-Pept/Dox NFs. (**D**) TEM micrographs of 1-Pept/Dox NFs. Bar, 200 nm. (**E**) TEM micrographs of 1-Pept/Dox NFs after treating with 5.0 U/mL Tps. Bar, 200 nm. (**F**) Circular dichroism spectra of 1-Pept/Dox NFs. (**G**) Dynamic frequency scanning of 1-Pept/Dox NFs. (**H**) Fluorescence spectra of Dox solution incubated with varying concentrations of 1-Pept for 30 min, with the insert displaying the plot of emission intensities at 590 nm against concentrations of 1-Pept. (**I**) Optical images of 1-Pept/Dox NFs, before and after being treated with PBS or 2 M NaCl

In order to further investigate the potential application of 1-Pept in the treatment of colon cancer, the commonly used chemotherapeutic medication Dox with positive charges was employed to co-assemble with 1-Pept, resulting in the formation of 1-Pept/Dox NFs through electrostatic interactions between Dox and the aspartic acid in the peptide (refer to Fig. 2A). For 1-Pept/Dox NFs, 1-Pept not only served as a drug carrier but also potentially played a synergistic role with Dox. The co-assembly behavior of 1-Pept and Dox was first evaluated. 1-Pept (1.0 wt%) co-assembled with Dox (0.1 eq) into 1-Pept/Dox nanofibrillar hydrogels with red color, which could be manipulated to write “HUCM” with a syringe needle (Fig. 2B). As shown in Fig. 2C, 1-Pept/Dox NFs displayed a decreased MGC (0.5 wt%) compared with 1-Pept (0.8 wt%). Correspondingly, the value of CAC of 1-Pept/Dox NFs (115 µM) was also lower than that of 1-Pept NFs (185 µM). TEM measurement indicated that 1-Pept co-assembled with Dox and formed slightly denser nanofibers compared with individual 1-Pept, suggesting the facilitation of Dox on the assembly process (Fig. 2D). Upon addition of Tps to the 1-Pept/Dox solution, a dense network of nanofibers was observed, underscoring the crucial role of Tps in the assembly process (Fig. 2E). Interestingly, CD spectra indicated an analogous secondary structure of β-sheet, a positive peak near 195 nm and a negative peak at 216 nm (Fig. 2F). Dynamic frequency scanning indicated that both values of G’ and G’’ of 1-Pept/Dox NFs were hardly affected by frequency variations, signifying the good mechanical properties of 1-Pept/Dox NFs (Fig. 2G).

To validate the interactions between 1-Pept and Dox, a series of 1-Pept solutions at various concentrations were incubated with 20 µg/mL Dox solution for fluorescent spectra analysis. As shown in Fig. 2H, the fluorescence signal intensity of Dox was decreased along with the increase of the concentration of 1-Pept. The quenching of Dox fluorescence may be attributed to the aggregation of Dox, induced by the assembly between Dox and 1-Pept. Notably, the addition of 2 M NaCl in 1-Pept/Dox NFs promoted the transition from gelation to solution within 3 min, confirming the existence of the electrostatic interaction between 1-Pept and Dox (Fig. 2I).

To investigate the intracellular Tps responsiveness, HT29 cells were treated with 1-Pept/Dox NFs for TEM observation. As shown in Fig. S13A, the slender nanofibers observed in HT29 cell lysates are likely the result of intracellular re-assembly of 1-Pept. To test this hypothesis, HT29 cells were pre-incubated with a Tps inhibitor (AEBSF, 150 µM) for 2 h and incubated with 1-Pept/Dox NFs for 12 h. TEM analysis revealed a minimal presence of nanofibers in HT29 cells treated with AEBSF (Fig. S13B), suggesting the involvement of Tps in the re-assembly process.

### Promoted 1-Pept/Dox NFs-mediated chemotherapeutic activity

**Fig. 3:**
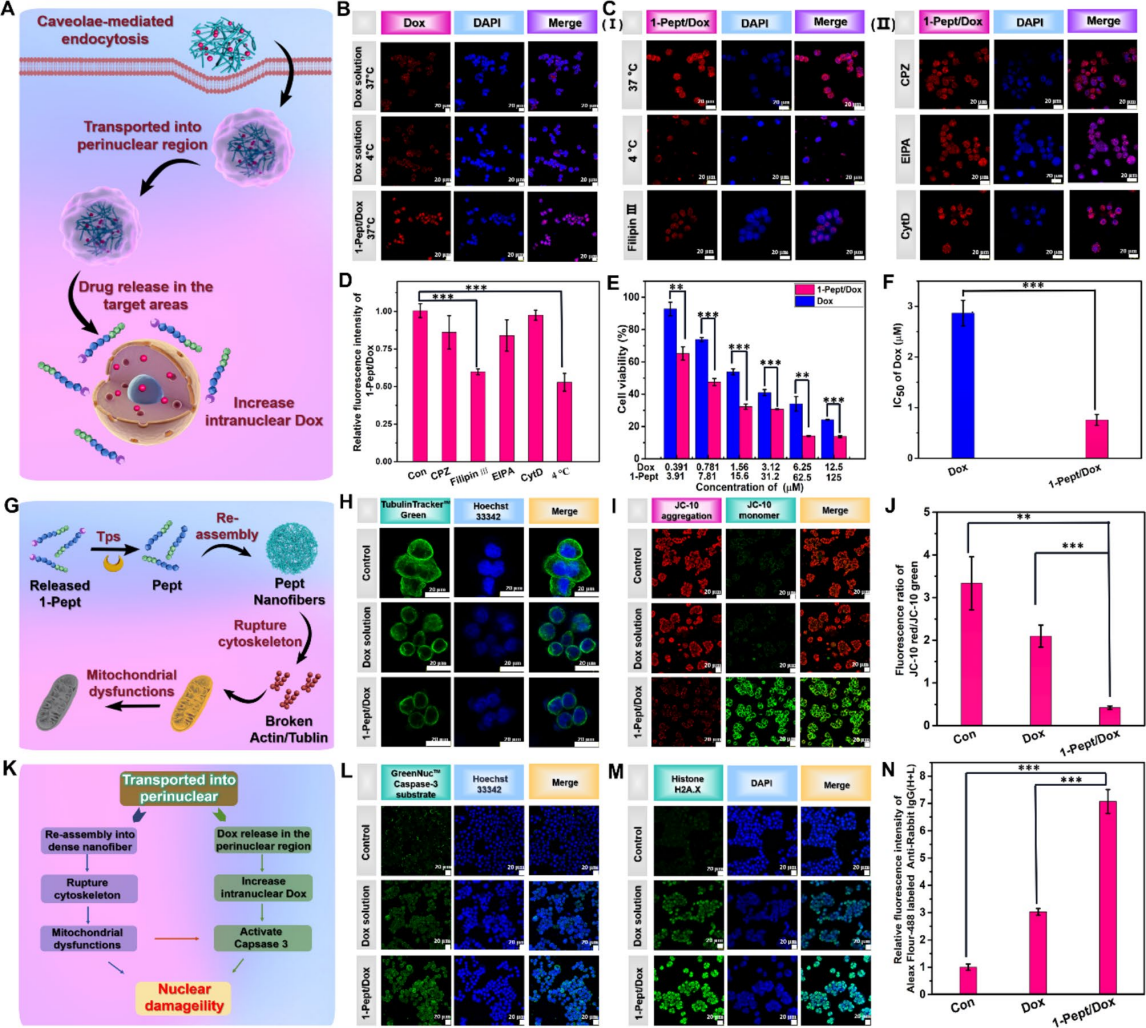
1-Pept/Dox NFs with cascading transformations to promote chemotherapy against colon cancer cells. (**A**) Diagram of 1-Pept/Dox NFs perinuclear transportation for increasing drug concentration in the target area. (**B**) Cellular uptake of 1-Pept/Dox NFs and Dox solution in HT29 cells. (**C**) CLSM observations of HT29 cells, treated with 1-Pept/Dox NFs at 37 °C–4 °C; CLSM images of HT29 cells, pretreated with endocytic inhibitor for 2 h and incubated with 1-Pept/Dox NFs for 6 h. Red, 1-Pept/Dox NFs; Blue, DAPI stained nuclei. Bar, 20 μm. (**D**) Relative fluorescence intensities of 1-Pept/Dox NFs of HT29 cells in Fig. 3C. (**E**) Cell viability of HT29 cells after treatment of 1-Pept/Dox NFs or free Dox for 48 h. (**F**) IC_50_ values of 1-Pept/Dox NFs and free Dox. (**G**) Schematic diagram of enzymatic reassembly of 1-Pept/Dox NFs, which was used to destroy cytoskeleton structure, inducing mitochondrial dysfunction. (**H**) CLSM images of intracellular tubulin fluorescence signals after being treated with 1-Pept/Dox NFs or Dox for 12 h. Green, Tubulin Tracker^™^ Green-stained tubulin; Blue, Hoechst 33,342-indicated nucleus. Bar, 20 μm. (**I**) CLSM images of HT29 cells for indicating ΔΨ_m_ with JC-10 detection kit. Red, JC-10 aggregates, representing a high ΔΨ_m_; Green, JC-10 monomer, indicating a low ΔΨ_m_. Bar, 20 μm. (**J**) Fluorescence ratio of JC-10 red/JC-10 green of HT29 cells in Fig. 3I. (**K**) Schematic diagram of 1-Pept/Dox NFs induced nuclear damage through intracellular cascade actions. (**L**) CLSM images of HT29 cells, used for tracking intracellular caspase-3 expression. Green, GreenNuc™ caspase-3 substrate; Blue, Hoechst 33,342-indicated nucleus. Bar, 20 μm. (**M**) Visualization of 1-Pept/Dox NFs or Dox-induced DNA damage by immunofluorescence staining. Green, Histone H2A.X foci; Blue, DAPI-stained nucleus. Bar, 20 μm. (**N**) Relative fluorescence intensities of Aleax Fluor 488-conjugated Anti-Rabbit IgG(H + L) in 1-Pept/Dox NFs treated HT29 cells in Fig. 3M

To investigate the capability of 1-Pept/Dox NFs in anticancer treatment, we selected colon cancer cells HT29 with overexpressed Tps for in vitro anticancer activity evaluation. Since intracellular internalizations were crucial for drug efficacy, the cellular uptake of 1-Pept/Dox NFs in HT29 was then explored (Fig. 3A). After an incubation of 6 h, the fluorescence signal intensity of Dox in 1-Pept/Dox NFs-treated cells was 2.4 fold than that of free Dox solution at 37 °C, suggesting the reinforced cellular uptake of 1-Pept/Dox NFs (Fig. 3B). To verify the mechanism of drug internalization, 6 h of incubation with free Dox solution at 4 °C was also performed. No differences were found in terms of cellular uptake of free Dox solution, indicating that the transport form by which drugs entered cells was passive diffusion (Fig. 3B) [50]. After 6 h incubation with 1-Pept/Dox NFs at 4 °C, the intracellular fluorescence signal of Dox was significantly reduced compared with that of cells receiving treatment of 1-Pept/Dox NFs at 37 °C (Fig. 3C). The differential uptake of 1-Pept/Dox NFs between 37 °C and 4 °C was potentially attributed to the facilitation of endocytosis-mediated cellular transport of the co-assembled nanofibers in 1-Pept/Dox NFs. Different endocytic pathways govern the fate of nanomedicines inside cells. For example, nanomedicines mainly enter lysosomes and degrade *via* clathrin-mediated endocytosis [51]. Caveolae-mediated endocytosis is preferable for drug internalization due to its capacity to bypass the lysosome, escape from degradation and further promote perinuclear transportation [52, 53]. To investigate the endocytic pathways of 1-Pept/Dox NFs, the cells were pretreated with various endocytosis inhibitors for further CLSM observations. The cellular fluorescence intensities of Dox slightly decreased in CPZ, EIPA and CytD-treated groups. Notably, the fluorescence signal intensity of Dox in 1-Pept/Dox NFs decreased significantly after receiving the treatment of Filipin III (Fig. 3C). After calculation, the fluorescence intensity of Filipin III-treated cells was 0.59 times that of untreated cells (Fig. 3D), indicating the caveolae-mediated endocytosis for 1-Pept/Dox NFs in HT29 cells. Recent research demonstrated that caveolae-mediated endocytosis could protect nanomedicines from the degradation of lysosomes [52, 53]. To validate it, the co-localization of 1-Pept/Dox NFs with lysosome was investigated with Lyso-Tracker Green under CLSM. As shown in Fig. S14, the red fluorescence of 1-Pept/Dox NFs almost did not overlap with the lysotracker signals. Over the duration of the experiment, though the fluorescence signals of 1-Pept/Dox NFs enhanced, they kept separated from the lysosome signal. These results further proved that caveolae-mediated endocytosis of 1-Pept/Dox NFs could efficiently avoid the lysosomal transport pathway, which was beneficial for more drugs reaching the perinuclear region, thus further increasing the concentration of Dox in the nucleus.

We speculated that the cellular uptake elevation could lead to cytotoxicity enhancement. Thus, the cytotoxicity assay was carried out to verify it. Upon the treatment of Dox solution and 1-Pept/Dox NFs for 48 h, the cell survival rates were reduced in a dose-dependent manner (Fig. 3E). Moreover, the cell viability was reduced obviously in HT29 cells treated with 1-Pept/Dox NFs compared with the free Dox group at the same concentration. Further calculations indicated that the IC_50_ value of Dox was 2.8 µM in free Dox solution, which was 3.7 fold higher than that of Dox in 1-Pept/Dox NFs (Fig. 3F). Data analysis and IC_50_ calculation indicated that the IC_50_ of 1-Pept in 1-Pept/Dox NFs was 7.5 µM, which was much lower than the IC_50_ value of 1-Pept used alone (134 µM). The combination index (CI) for 1-Pept/Dox NFs was 0.32, suggesting that 1-Pept promoted the cytotoxicity of Dox synergistically against HT29 cells. In addition, the inhibitory effects of 1-Pept/Dox on colon cancer cell lines (SW620, SW480, and T84) with high Tps expression [54, 55] were notably better than those treated with Dox alone (Fig. S15A-C). In contrast, there was almost no significant difference in cell survival rates of colon cancer cell line HCT-15 with low Tps expression [54] treated with 1-Pept/Dox and those treated with Dox alone (Fig. S15D). These results suggest that 1-Pept significantly enhances the anticancer activity of Dox in cell lines with high Tps expression.

The formation of intracellular nanofibers may be crucial for enhancing Dox cytotoxicity against cancer cells by destroying the cytoskeleton (Fig. 3G). Therefore, we investigated the morphological changes of the cytoskeleton in 1-Pept/Dox-treated cells using Alexa Fluor 633 phalloidin for staining F-action filaments. As shown in Fig. S16, the fluorescent intensity of Alexa Fluor 633 phalloidin in Dox-treated cells exhibited a certain degree of decrease compared to untreated cells. It was worth noting that the fluorescence intensity was significantly decreased in 1-Pept/Dox NFs-treated cells, indicating a severe structural failure of F-actin. Furthermore, microscopic deformation and fragmentation of intracellular microtubules were explored by Tubulin Tracker™ Green-stained cells under CLSM observation. As shown in Fig. 3H, the untreated cells exhibited well-defined microtubules, whereas fewer well-structured microtubules were observed in Dox and 1-Pept/Dox NFs-treated cells. By comparison, the most well-defined microtubules could not be observed in 1-Pept/Dox NFs-treated cells, which was plausibly attributed to intracellular reassembly catalyzed by overexpressed Tps.

Increasing data suggested that the destruction of the cytoskeleton could induce mitochondrial dysfunction, activate the apoptosis pathway, and further increase the permeability of the nuclear envelope [56]. Subsequently, we examined the changes in ΔΨ_m_ with a JC-10 assay kit to quantify the red/green fluorescence ratio. As shown in Fig. 3I, the red fluorescence signal intensities decreased, and the green fluorescence signal intensities increased in 1-Pept/Dox NFs-treated cells compared with that of the untreated and Dox group. Further calculation indicated that the red/green fluorescence ratio of 1-Pept/Dox group was 7.8 and 4.8 times lower than that of the control and the Dox group, exhibiting a significant decrease of ΔΨ_m_ after treatment of 1-Pept/Dox NFs (Fig. 3J). We explored the activation of apoptosis in HT29 cells mediated by decreased mitochondrial membrane potential (Fig. 3K). GreenNuc™ Caspase-3 assay kit was used to explore the intracellular caspase-3 activity under CLSM. As shown in Fig. 3L, the fluorescent signal intensity of GreenNuc™ caspase-3 substrate in non-drug treated cells and Dox-treated cells was low, indicating the weak expression of caspase-3. In comparison, the corresponding result of the 1-Pept/Dox NFs-treated cells exhibited a stronger fluorescent signal, suggesting a higher level of caspase-3. After calculation, the fluorescence signals of 1-Pept/Dox NFs group were 1.7 fold than in the Dox group, indicating the role of 1-Pept in promoting apoptosis and enhancing cytotoxicity.

Given that Dox exerts its anti-cancer effects by inserting into DNA and further inhibiting nucleic acid synthesis [57], we investigated 1-Pept/Dox-induced DNA damage by observing nuclear damage marker (Histone H2A.X) expression level. As shown in Fig. 3M, the control group without any treatment exhibited almost no green fluorescence signal, whereas the Dox-treated cells showed bright green fluorescence intensity, indicating DNA damage. Intriguingly, 1-Pept-treated cells also gave a comparatively green fluorescence signal, suggesting cytoskeleton disruption triggered DNA damage. In all groups, 1-Pept/Dox-treated cells exhibited the strongest fluorescence signal intensity, which was 7.0 and 2.3 fold higher than that of the blank control and the Dox group, suggesting its synergistic role in promoting DNA damage (Fig. 3N).

Collectively, 1-Pept/Dox NFs enhanced the cytotoxicity of Dox *via* multiple mechanisms. Caveolae-mediated endocytosis could bypass the lysosome and increase the perinuclear drug concentration to further increase the drug concentration in the nucleus. 1-Pept could activate apoptosis by disrupting the cytoskeleton and destroying mitochondrial function, and benefit Dox to enter the nucleus as the increase of nuclear envelope permeability along with the occurrence of apoptosis. The 1-Pept could activate apoptosis and induce DNA damage, further enhancing the cytotoxicity of Dox.

#### 1-Pept/Dox NFs boosted sustained release of dox in vitro and prolonged drug duration in vivo

**Fig. 4:**
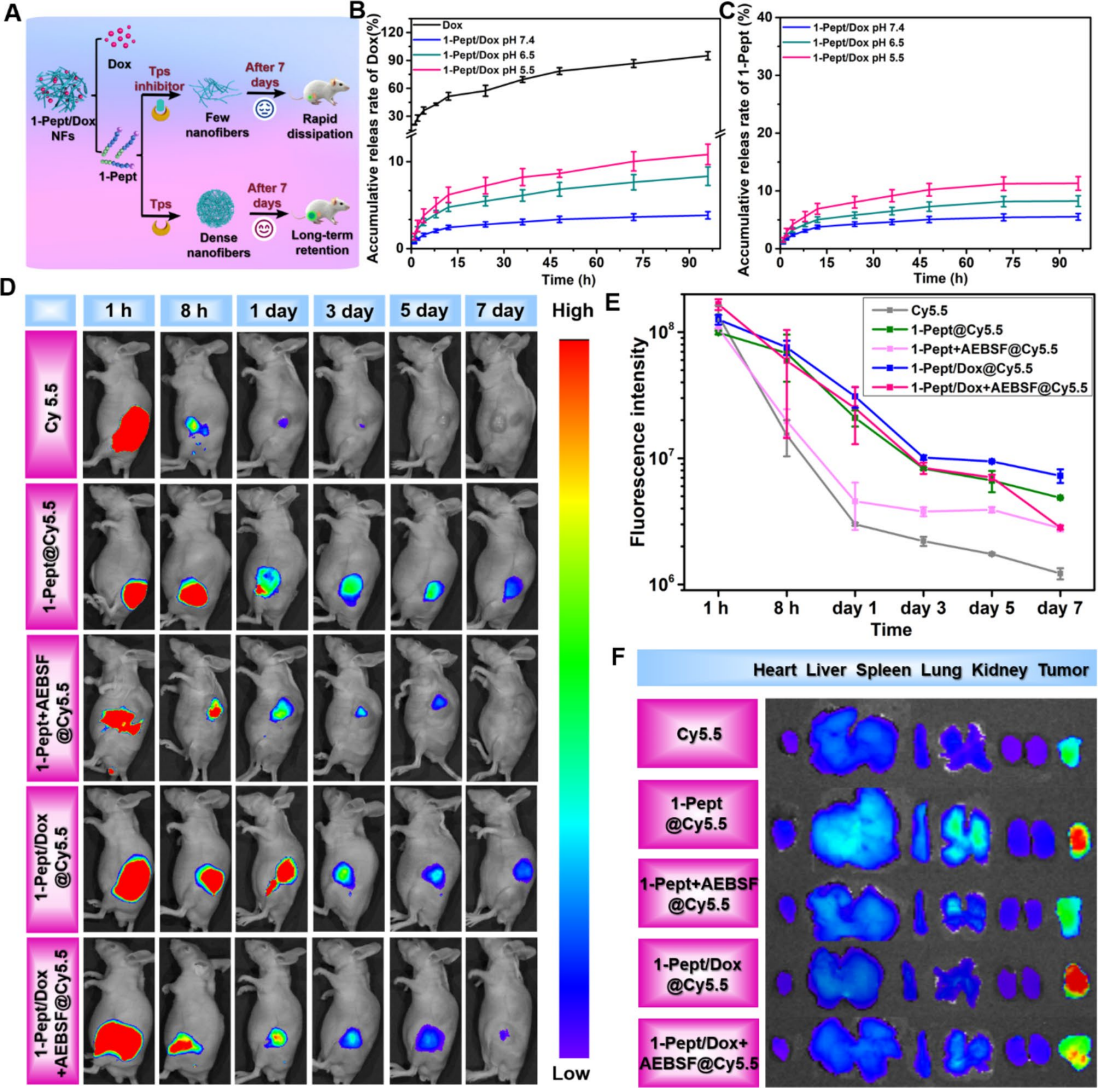
Characterizations of 1-Pept/Dox NFs for sustained-release of Dox in vitro and enhanced drug duration in vivo. (**A**) Schematic diagram of drug release and body retention of 1-Pept/Dox NFs. (**B**) Accumulative release rates of Dox from 1-Pept/Dox NFs at varying pH conditions and Dox solution served as control. (**C**) Accumulative release rates of 1-Pept from 1-Pept/Dox NFs at varying pH conditions. (**D**) Live imaging of HT29-xenografted mice, respectively treated with Cy5.5 solution, 1-Pept@Cy5.5, 1-Pept + AEBSF@Cy5.5, 1-Pept/Dox@Cy5.5, and 1-Pept/Dox + AEBSF@Cy5.5. (**E**) Quantification of fluorescence intensity of HT29-xenografted mice at different time points after receiving different formulation treatments. (**F**) Fluorescence images of isolated tumor and major organs after administration of 1 day

The in vitro release performances of Dox from free Dox solution and 1-Pept/Dox NFs were evaluated (Fig. 4A). As shown in Fig. 4B, the Dox solution exhibited a burst release profile, with a cumulative release of Dox, reaching 51% for 12 h. To further explore the drug’s release mechanism, a Ritger-Peppas model was employed. As shown in Table S1, the exponential factor (n) of the free Dox solution was 0.35, indicating the release of Dox from the free Dox solution followed the Fickian diffusion mechanism. The cumulative release rates of Dox from 1-Pept/Dox NFs were less than 10% with 12 h in all pH ranges, which was significantly lower than that of free Dox solution, suggesting 1-Pept/Dox NFs possess a sustained release profile. We investigated the pH-responsive release profiles under different pH (7.4, 6.5, and 5.5) conditions. In a neutral dissolution medium, the cumulative release rate of Dox reached 2.7% at 24 h. Dox was released form 1-Pept/Dox NFs with accelerated speeds at an acidic dissolution medium. The cumulative release rates of Dox form 1-Pept/Dox NFs were 5.5% and 7.2%, respectively, in pH 6.5 and 5.5 dissolution medium at the 24 h point, which were significantly greater than that of the released rate in pH 7.4, suggesting the acidity-responsive release performance for 1-Pept/Dox NFs (Fig. 4B). A further Ritger-Peppas model fitting within 12 h was applied, and the n-value of 1-Pept/Dox NFs at pH 7.4, 6.5, and 5.5 was 0.53, 0.54, and 0.58, demonstrating the non-Fickian diffusion of Dox released from 1-Pept/Dox NFs. Consistently, the cumulative release rates of 1-Pept form 1-Pept/Dox NFs at pH 7.4 were also slower than those at pH 6.5 and 5.5 (Fig. 4C). The calculated n values of 1-Pept were 0.54, 0.55, and 0.58 at pH 7.4, 6.5, and 5.5, respectively, which were greater than 0.43, suggesting a non-Fickian diffusion for 1-Pept released from 1-Pept/Dox NFs (Table S2). In general, 1-Pept/Dox NFs exhibited an acid-responsive profile, which was potentially attributed to the protonation of carboxylic acid groups of 1-Pept and weakened electrostatic interaction between 1-Pept and Dox.

As a drug reservoir, it is vital for 1-Pept/Dox NFs to have favorable injectability and stability in terms of in vivo applications. Considering this, a fluorescence dye Cy5.5 was loaded in 1-Pept/Dox NFs for observing the body retention of this system. As shown in Fig. 4D, there was no fluorescence signal of Cy5.5 solution in mice, 1-day post-injection, implying the rapid clearance of Cy5.5 solution in the subcutaneous area. By comparison, the signal of Cy5.5 for 1-Pept@Cy5.5 and 1-Pept/Dox@Cy5.5 groups could still be observed in the subcutaneous area after 7 days of subcutaneous injection, suggesting the excellent in vivo retention ability of 1-Pept and 1-Pept/Dox NFs (Fig. 4D). In addition, the fluorescence signal intensity of Cy5.5 for the 1-Pept/Dox@Cy5.5 group was 1.4 times of 1-Pept@Cy5.5 group on day 7, signifying the facilitation of Dox on the assembly process. To verify that the involvement of Tps in the tumor microenvironment could enhance the stability of the system in vivo, Tps inhibitor AEBSF was loaded into 1-Pept@Cy5.5 and 1-Pept/Dox@Cy5.5 to obtain 1-Pept + AEBSF@Cy5.5 and 1-Pept/Dox + AEBSF@Cy5.5, respectively. In vivo imaging results revealed that the fluorescence signal intensity of both 1-Pept + AEBSF@Cy5.5 and 1-Pept/Dox + AEBSF@Cy5.5 was significantly lower than those groups without Tps inhibitor AEBSF, implying the probable contribution of Tps to the enhanced body retention in the tumor microenvironment (Fig. 4E). As shown in Fig. 4F, the ex vivo fluorescence signals of tumors in the 1-Pept@Cy5.5 and 1-Pept/Dox@Cy5.5-treated group were significantly stronger than the free Cy5.5-treated group. The fluorescence signal of tumors was greater than the major organs, especially the heart, kidney, and spleen in the 1-Pept/Dox@Cy5.5-treated group. Furthermore, the fluorescence intensity of the tumor of the 1-Pept/Dox + AEBSF@Cy5.5 group was decreased compared with the 1-Pept/Dox@Cy5.5 group, confirming that the inhibition of AEBSF for Tps could interfere with 1-Pept-mediated re-assembly. In brief, these results demonstrated that 1-Pept/Dox NFs could achieve long-term retention at the tumor site under the catalysis of overexpressed Tps in the tumor microenvironment.

### In vivo efficacy of 1-Pept/Dox NFs to HT29 xenografted tumor in mouse

**Fig. 5:**
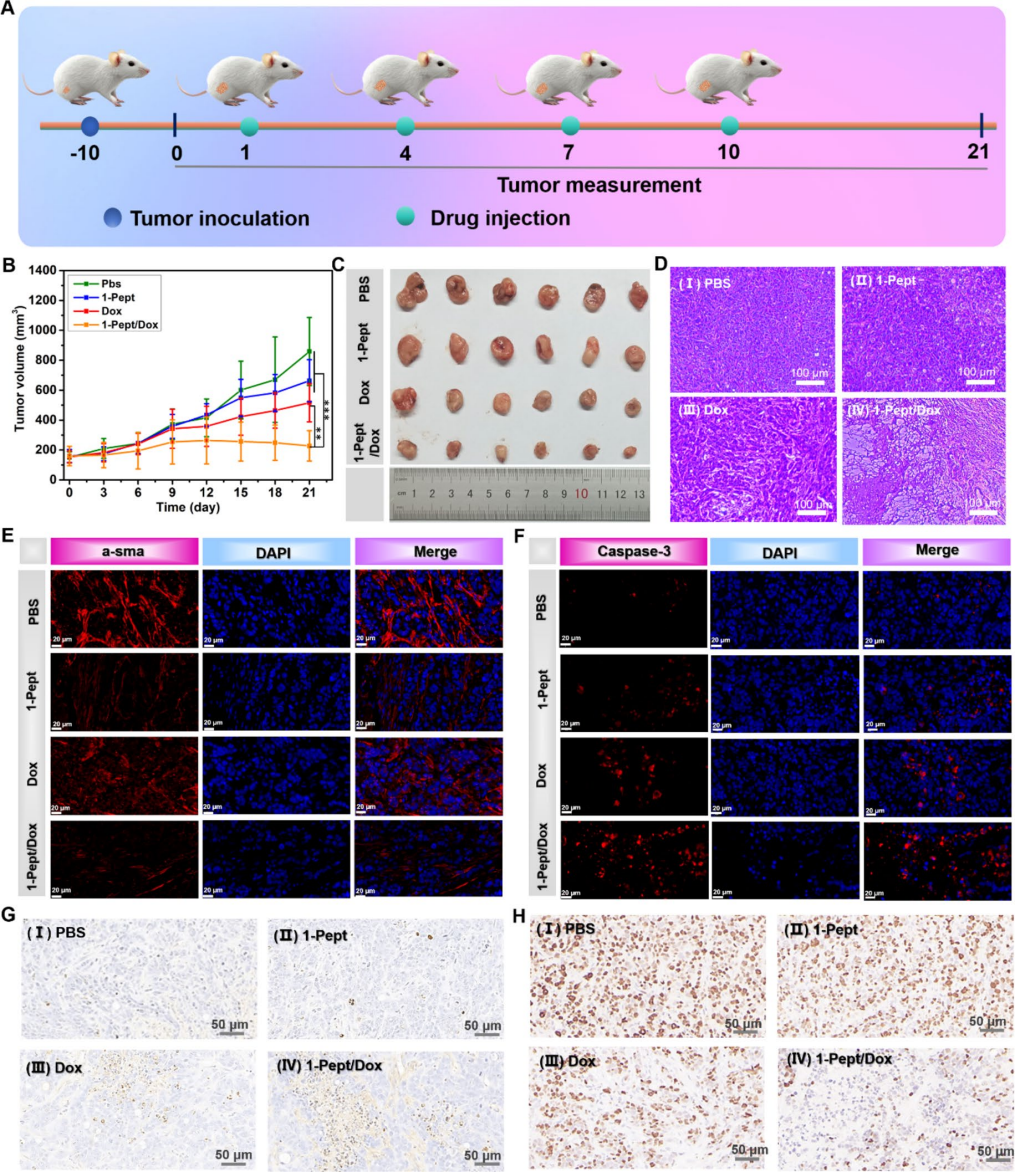
Examinations on 1-Pept/Dox NFs anti-tumor activity in vivo. (**A**) A diagrammatical schedule of tumor inoculation, drug injection, and tumor measurement. (**B**) Tumor growth curves in HT29-xenografted mice after receiving PBS, 1-Pept, Dox, and 1-Pept/Dox NFs treatments. (**C**) Photographs of isolated tumors from various formulations-treated mice on the day 21st. (**D**) H&E-stained tumors, which received various treatment formulations. Bar, 100 μm. (**E**) Fluorescence immunoassay of α-SM actin in different groups-treated tumor tissues. Red, α-SM actin; Blue, DAPI. Bar, 20 μm. (**F**) Fluorescence immunoassay of caspase-3 expressions in different-treated tumor tissues. Red, caspase-3; Blue, DAPI. Bar, 20 μm. (**G**) TUNEL staining images of tumor tissues. Bar, 50 μm. (**H**) Ki67-staining images of tumor tissues. Bar, 50 μm

After investigating the encouraging results of in vitro performance, we studied the in vivo antitumor efficacy of 1-Pept/Dox NFs with HT29-xenografted tumor model in nude mice. When the tumor volume reached 100 mm^3^, 1-Pept/Dox NFs were subcutaneously injected by the peritumoral site into the right flank of the nude mice (Fig. 5A). Meanwhile, the PBS group, free Dox solution, and 1-Pept were designed as controls. By observing dynamic changing curves of tumor volume, it could be found that the PBS group gave the fastest tumor growth within the measurement time range. In contrast, subcutaneous injection of 1-Pept/Dox NFs gave a significant tumor growth inhibition, indicating the superb antitumor efficacy of 1-Pept/Dox NFs. In comparison, injection of Dox or 1-Pept exhibited a feeblish inhibition on tumor proliferation (Fig. 5B). After calculation, the tumor volume growth rates on the 21st day were 5.7, 4.2, 3.3, and 1.5 for PBS, 1-Pept, Dox, and 1-Pept/Dox NFs groups, respectively (Fig. 5C). After the end of treatment, we characterized the isolated tumor tissues from the nude mice for hematoxylin-eosin (H&E) staining. As shown in Fig. 5D, the cell densities of the 1-Pept and Dox groups were slightly lower than that of the PBS group, which was consistent with the results of relative tumor volumes, further proving its poor tumor inhibition effects. The tumor tissues of 1-Pept/Dox NFs groups exhibited the lowest cell density and showed extensive necrosis, suggesting the unique role of 1-Pept in enhancing the anti-cancer efficacy of Dox. Therefore, 1-Pept/Dox NFs exhibited efficient tumor suppression efficacy, which was plausibly attributed to the enzymatic transformations in the tumor microenvironment.

Next, we study the change of actin filament in tumor tissues by immunofluorescence staining. The control group exhibited well-defined actin filaments with a bright red fluorescence signal of α-SM actin (Fig. 5E). In comparison, the α-SM actin in the Dox and the 1-Pept group showed a certain degree of destruction. Disorganized and broken actin filaments with faint red fluorescence signals could be seen in the tumor tissues of 1-Pept/Dox NFs, revealing the most severe actin structural damage after receiving treatment of 1-Pept/Dox NFs. After that, the tubulin structure changes in various groups-treated tumor tissues were investigated. As shown in Fig. S17, a well-formed labeled tubulin structure was found in the PBS group, whereas tubulin with moderate structural damage was observed in the 1-Pept and Dox groups, and the 1-Pept/Dox NFs-treated group displayed serious tubulin structural damage. These results indicated that 1-Pept/Dox NFs could efficiently induce cytoskeleton disruption.

The expression of caspase-3, a cell apoptosis activating factor, was detected using a GreenNuc™ caspase-3 activity assay kit. As shown in Fig. 5F, there was almost no red fluorescence signal of labeled caspase-3 in the control group, indicating the low-level activated caspase-3. In comparison, the 1-Pept or Dox groups showed increasing red fluorescence signal intensity, which was ascribed to the activation of caspase-3 after receiving different treatments. The fluorescence signal intensity in HT29 cells treated with 1-Pept/Dox NFs was greatly increased, which was 3.3 and 2.4 times than that in 1-Pept and Dox groups, confirming the highest expression level of activated caspase-3 in 1-Pept/Dox NFs group (Fig. S18).

TUNEL staining was employed to observe apoptotic cells in tumor tissues. As shown in Fig. 5G, 1-Pept/Dox NFs-treated tumor tissues exhibited massive TUNEL-positive cell expression, revealing that 1-Pept/Dox NFs could effectively induce HT29 cancer cell apoptosis. In comparison, fewer or almost no TUNEL-positive cells were found in PBS, Dox solution, or 1-Pept groups. Further quantitative analysis indicated that the percentage of apoptotic cells in 1-Pept/Dox NFs-treated tumor tissues was 5.4, 2.5, and 2.3 fold compared to that in blank control, 1-Pept, and Dox group (Fig. S19). Ulteriorly, we verified the tumor inhibition of 1-Pept/Dox NFs using immunohistochemical analysis of Ki67, a mark for indicating cell proliferation. As shown in Fig. 5H, Ki67 expression in the 1-Pept/Dox NFs group was lower than that of PBS, 1-Pept, and Dox groups, signifying the strong inhibiting effects on tumor proliferation. The percentage of Ki67-positive signals in tumor tissues were 70%, 60%, 45%, and 37% for PBS, 1-Pept, Dox, and 1-Pept/Dox NFs groups, respectively (Fig. S20).

Collectively, these results indicated that 1-Pept/Dox NFs-mediated EISA could induce a cascade of effects including disruption of the cytoskeleton, mitochondrial dysfunction, and activation of caspase-3, causing greater damage to nuclear damage, thereby effectively ablating the tumor by the synergism of Pept NFs and Dox.

### Examinations of biocompatibility of 1-Pept/Dox NFs

**Fig. 6:**
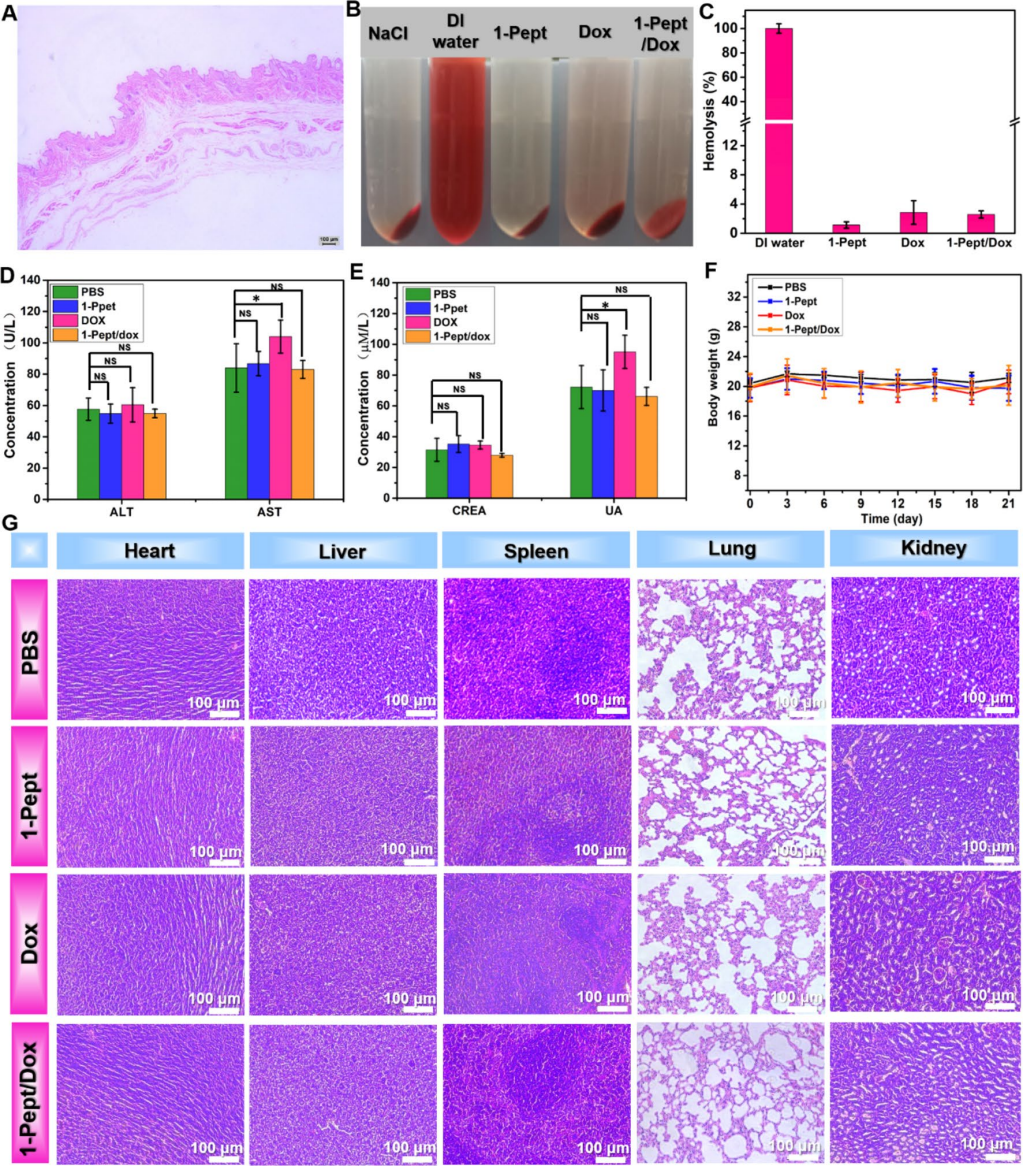
Biocompatibility evaluations of 1-Pept/Dox NFs. (**A**) HE staining image of the peripheral histiocyte of the injection site after 1-Pept/Dox NFs treatment. (**B**) Photographs of rabbit red blood cell suspensions after treatment with NaCl solution, DI water, 1-Pept NFs, Dox solutions, or 1-Pept/Dox NFs. (**C**) Hemolysis rates of rabbit red blood cells after treatment with DI water, 1-Pept NFs, Dox solutions, or 1-Pept/Dox NFs. (**D**) Determined parameters of hepatotoxicity of mice. (**E**) Determined parameters of nephrotoxicity of mice. (**F**) Body weight changes of mice. (**G**) Histological staining images of major organs of mice treated in different groups. Bar, 100 μm

With the anti-tumor efficacy confirmed in vivo, the biocompatibility of 1-Pept/Dox NFs was further accessed, and the tissue reactions of 1-Pept/Dox NFs were investigated. As shown in Fig. 6A, 1-Pept/Dox NFs-treated skin tissues exhibited almost no lymphocyte infiltration or fibrous capsules, which revealed that 1-Pept/Dox NFs did not cause any visual dermatological irritation or inflammation.

Next, we evaluated the hemolysis rate of 1-Pept/Dox NFs to evaluate the hemocompatibility. As shown in Fig. 6B, no obvious hemolysis phenomena for 1-Pept, Dox, and 1-Pept/Dox NFs groups were observed. Quantitatively, the hemolysis rates were 1.1%, 2.8% and 2.6% for 1-Pept, Dox, and 1-Pept/Dox NFs groups, respectively, which were all below 5%, showing a good hemocompatibility of this delivery system (Fig. 6C). Subsequently, the hepatotoxicity of 1-Pept/Dox NFs was assessed by measuring the liver function biomarkers, including alanine transaminase (ALT) and aspartate transaminase (AST). The measured values of the 1-Pept/Dox NFs group exhibited no differences compared with that of control groups, which were within the normal value ranges, suggesting no obvious hepatotoxicity from 1-Pept/Dox NFs (Fig. 6D). The kidney function parameters including creatinine (CREA) and uric acid (UA) were also evaluated. CREA and UA levels were also within the normal value ranges for all groups, indicating no significant nephrotoxicity from the system (Fig. 6E). In addition, the mice’s body weights didn’t exhibit significant variation after giving different formulations treatments (Fig. 6F).

Finally, we processed to evaluate the systemic toxicity of the delivery system by histological analysis. As revealed in Fig. 6G, the main organs, including the heart, liver, spleen, lung, and kidney, were stained with hematoxylin-eosin. Compared with the PBS group, no obvious morphological changes in the free Dox solution group, 1-Pept group, or 1-Pept/Dox NFs group were observed, proving the good biocompatibility of the drug delivery system. Collectively, these results further revealed that 1-Pept/Dox NFs not only exhibit excellent anticancer efficacy but also possess satisfactory biocompatibility in vivo.

## Materials and methods

### Materials

2-Chlorotrityl chloride resin and Fmoc-Amino acid were purchased from Nanjing Peptide Biochemical Ltd (Nanjing, China). Doxorubicin (Dox) was purchased from Yuanye Biological Science and Technology Ltd (Shanghai, China). Chlorpromazine (CPZ) was the product of Macklin Biochemical Ltd (Shanghai, China). 5-(Ethylisopropyl) Amiloride (EIPA), 4-(2-Aminoethyl) benzenesulfonyl fluoride hydrochloride (AEBSF), and trypsin (Tps) were obtained from Aladdin Reagent Corporation (Shanghai, China). Filipin III and cytochalasin D (Cyt D) were GlpBio Technology (USA) products. Alexa Fluor™ 633 and Tubulin Tracker™ Green were obtained from Thermo Fisher (USA). Histone H2A.X was obtained from Proteintech (Wuhan, China). Thiazolyl blue tetrazolium bromide (MTT), DMEM and 1640 medium were products of HyClone (USA). Fetal bovine serum (FBS) was the product of Grand Island Biological Company (USA). All cells were acquired by the American Type Culture Collection.

### Syntheses and purifications of 1-Pept

1-Pept was obtained by solid-phase peptide synthesis technology (SPPS), and the amino acids were sequentially connected to 2-chlorotrityl chloride resin until the sequence was completed. The compound was cleaved from the resin using TFA. After washing with ether three times, the compound was lyophilized to get a crude product. The samples were later purified by preparative-scale HPLC and characterized by ^1^H-NMR, TOF-MS, and HPLC. The purity of the obtained 1-Pept was 96%, which could be used in the following experiments.

### Trypsin-catalyzed conversion of 1-Pept

First, the Tps-catalyzed conversion rate of 1-Pept was investigated. 1-Pept (1 mg/mL) was dissolved in PBS solution, and the pH was modulated to a neutral condition. After that, Tps (1 U/mL) was added to the solution to trigger the conversion of 1-Pept (NapFFGYKCD) into Pept (NapFFGYK). At fixed time points, 50 µL 1-Pept solution was taken out and mixed with 150 µL methanol to obtain the mixed solution. Finally, the mixed solution was analyzed with HPLC-MS to investigate the conversion rate.

The enzyme-directed assembly behavior of 1-Pept was subsequently investigated. 1-Pept was dissolved in PBS solution, and the pH was adjusted to neutral condition. The formation of 1-Pept nanofibrillar hydrogels was monitored *via* a vial inversion method. 1-Pept/Tps NFs were obtained after mixing Tps (5 U/mL) with 1-Pept solution. The MGC of 1-Pept NFs and 1-Pept/Tps NFs could be determined when the samples could not flow.

The CAC was measured using a Malvern Zetasizer. 1-Pept (1.0 wt%) and 1-Pept/Tps (1.0 wt%, 5 U/mL) were prepared and diluted to solution with different concentrations. The light scattering intensity (Kcps) was obtained at 632.8 nm, 25 °C. The CAC values were obtained from the intersection point of two linear lines of Kcps against 1-Pept concentration.

The microscopic morphology of 1-Pept NFs (1.0 wt%) and 1-Pept/Tps NFs (1.0 wt%, 5 U/mL) were fabricated and diluted to 0.5 mg/mL. Then the solutions were stained with phosphotungstic acid and further observed under JEOL JEM1200EX.

For CD spectra, 1-Pept NFs and 1-Pept/Tps NFs were prepared according to the above process. Then the samples were diluted to 0.2 mg/mL and analyzed by circular dichroism spectroscopy (JASCO J-1500) from 400 nm to 190 nm.

The rheological experiment was recorded by a TA DHR-2 rheometer (Gap distance: 0.3 mm, Diameter: 60 mm) to investigate the mechanical properties of 1-Pept NFs and 1-Pept/Tps NFs. For dynamic time scanning, the G’ and G” of 1-Pept NFs and 1-Pept/Tps NFs were recorded within 60 min (6.28 rad/s frequency, 0.1% strain). Dynamic strain scanning was obtained from 0.1 to 100% strain at a specific frequency (6.28 rad/s). Dynamic frequency scanning was analyzed at 0.1% strain with a frequency from 0.1 to 100%.

### Preparation and characterizations of co-assembled 1-Pept/Dox NFs

1-Pept (1.0 wt%) was dissolved in PBS solution and mixed with 0.1 equivalents of Dox to obtain 1-Pept/Dox NFs (1.0 wt%/0.1 eq), and the solutions were kept at 25 °C to obtain 1-Pept/Dox NFs. The MGC, CAC, microscopic morphology, CD analysis, and viscoelasticity were tested according to the above procedures. The intermolecular force between 1-Pept and Dox was examined with fluorescent spectroscopy. Simply, Dox solution (20 µg/mL) was incubated with different concentrations (125, 62.5, 31.3, 15.6, 7.81, and 3.90 µg/mL) of 1-Pept solutions, and the fluorescent spectrum of the sample was recorded at a fixed excitation wavelength of 590 nm. Ulteriorly, the electrostatic interactions between 1-Pept and Dox were explored by adding sodium chloride solution (2 M).

### Measurement of cellular uptakes of 1-Pept/Dox NFs

A CLSM was used to detect the cellular uptakes of 1-Pept/Dox NFs in HT29 cells. HT29 cells were inoculated in laser confocal culture plates and treated with 1-Pept/Dox NFs (125 µM/12.5 µM) at 37 °C–4 °C for 6 h. With 4% formaldehyde treatments, the cells were treated with DAPI and recorded under a CLSM. Meanwhile, equivalent free Dox was used as a control.

To examine the endocytosis mechanism of 1-Pept/Dox NFs, HT29 cells were preincubated with different endocytosis inhibitors for 2 h. Co-culture the cells with 1-Pept/Dox (125 µM/12.5 µM) for 6 h and give an observation under CLSM to evaluate the fluorescent intensity of Dox. The following endocytosis inhibitors were chosen: CPZ (30 µM) for inhibiting clathrin-mediated endocytosis; Filipin III (0.5 µg/mL) for repressing caveolae-dependent endocytosis; EIPA (50 µM) and CytD (10 µM) for inhibiting microphagocytosis, and phagocytosis-mediated cellular uptake, respectively.

To investigate the involvement of the lysosome-mediated endocytosis pathway in intracellular drug transport, HT29 cells were co-cultured with 1-Pept/Dox (125 µM/12.5 µM) for 1–6 h, accompanied by incubation with Lyso-Tracker Green at a concentration of 75 nM for 30 min. Then, the cells were treated with Hoechst 33,342 for CLSM observation.

HT29 cells were pre-incubated with Tps inhibitor (AEBSF, 150 µM) for 2 h to confirm the involvement of Tps in the intracellular reassembly process. Then, the cells were incubated with 1-Pept/Dox (125 µM/12.5 µM) and 150 µM AEBSF for 12 h. Subsequently, the cells were washed with PBS buffer and treated with cell lysis buffer. After that, the samples were observed by TEM.

### Examinations of cytotoxicity of 1-Pept/Dox NFs against colonic cancer cell

The colonic cancer cell line HT29 was selected to explore the cytotoxicity of 1-Pept/Dox NFs against colonic cancer cells. First, the intracellular Tps expressions of HT29 cells were investigated. The human normal colonic epithelial cells NCM460, human melanoma cells A375, and human cervical cancer cells Hela were used as controls. The above cells were incubated at the cell incubator and treated with cell lysis buffer. Subsequently, the samples were tested with a quantitative determination kit for trypsin activity.

HT29 cells were incubated in a 96-well microplate and cultured with 1-Pept/Dox NFs for 48 h. The concentrations of Dox were between 12.5 and 0.391 µM, and 1-Pept were between 125 and 3.91 µM. Then, a fixed volume of 20 µL MTT (5 mg/mL) was added to the wells for co-incubation. After 4 h, the generated formazan could be found. After dissolving in DMSO, the absorbance values were recorded at 490 nm utilizing a microplate reader (Multiskan FC). By comparing the absorbance of the samples and the controls, the cell’s survival rates were calculated. The IC_50_ values were obtained by Origin 8.0 software. Simultaneously, the MTT assays for NCM460, SW620, SW480, and HCT-15 were performed using the aforementioned procedures.

### Mechanism investigation of 1-Pept/Dox NFs-caused cellular toxicity towards colonic cancer cell

To determine the influence of 1-Pept/Dox NFs on the actin, HT29 cells were inoculated in a laser confocal dish and co-cultured with 1-Pept/Dox (125 µM/12.5 µM) for 12 h. The cells were treated with formaldehyde and Triton X-100. Afterward, 1 mL Alexa Fluor™ 633 (33 nM) was incubated with cells for 20 min. Finally, the cells were treated with DAPI for 20 min and observed by a CLSM.

To confirm 1-Pept/Dox NFs induced perturbation towards tubulin, HT29 cells were co-cultured with 1-Pept/Dox (125 µM/12.5 µM) for 12 h. The cells were treated with 1X Tubulin Tracker™ Green working solution for 30 min. After that, the cells were incubated with Hoechst 33,342 for CLSM observation.

A mitochondrial membrane potential reagent kit was chosen to validate the mitochondrial dysfunction after drug treatment. HT29 cells were co-cultured with 1-Pept/Dox NFs (125 µM/12.5 µM) for 12 h. The cells were incubated with JC-10 reagent for 30 min. After treatment with PBS, the intracellular fluorescence was observed under a laser confocal microscope. The JC-10 monomers and aggregates were observed separately at excitation wavelengths of 515 and 585 nm.

After 1-Pept/Dox NFs treatment, the GreenNuc™ live cell caspase-3 activity detection kit was used to explore the cleaved caspase-3 level. HT29 cells were co-incubated with 1-Pept/Dox (125 µM/12.5 µM) for 12 h and incubated with GreenNuc™ caspase-3 substrate (5 µM) for 30 min. Subsequently, the cells were stained with Hoechst 33,342 for CLSM observation.

To evaluate the effects of 1-Pept/Dox NFs on the nucleus, HT29 cells were plated in a laser confocal dish, co-incubated with 1-Pept/Dox (125 µM/12.5 µM) for 12 h, and were treated with 4% formaldehyde and 0.1% Triton X-100. Then, the cells were treated with rabbit anti-histone H2A.X polyclonal antibody (Histone H2A.X) for 12 h. After that, the cells were cultured with Aleax Fluor 488-conjugated Anti-Rabbit IgG(H + L) for 4 h and observed using a CLSM.

### In vitro release of Dox from 1-Pept/Dox NFs and retention behavior of 1-Pept/Dox NFs in vivo

The in vitro drug release profile of 1-Pept/Dox NFs was conducted in a constant temperature vibrator (40 rpm, 37 °C). 1-Pept/Dox NFs (200 µL, 1.0 wt%/0.1 eq) were prepared and incubated with 200 µL PBS buffer (pH = 7.4, 6.5 and 5.5). At particular time points, 200 µL liquid supernatant was sucked out and further analyzed by HPLC. Simultaneously, 200 µL fresh PBS was added to maintain a fixed volume. Free Dox solutions were used as controls.

To study the retention behavior of hydrogels in vivo, a near-infrared (NIR) fluorescent dye Cy5.5 was loaded in various formulations to indicate the changes of the formations in vivo at specific time points. 1-Pept NFs and 1-Pept/Dox NFs were prepared according to the above procedures and were mixed with 20 µg/mL Cy5.5 to obtain 1-Pept@Cy5.5 NFs and 1-Pept/Dox@Cy5.5 NFs, respectively. For validating the indispensable role of Tps in tumor microenvironment, the Tps inhibitor 4-(2-Aminoethyl) benzenesulfonyl fluoride hydrochloride (AEBSF, 1 mg/mL) was treated with 1-Pept@Cy5.5 NFs and 1-Pept/Dox@Cy5.5 NFs to obtain 1-Pept + AEBSF@Cy5.5 NFs and 1-Pept/Dox + AEBSF@Cy5.5 NFs, respectively. The formulations were subcutaneously injected into the tumor-adjacent area of HT29 tumor-bearing nude mice. In vivo fluorescence signals could be watched at specific time points under a small animal in vivo imaging instrument. After 1 day post-subcutaneous injection, the major organs and tumors of nude mice were stripped and observed under the in vivo imaging instrument.

### Efficacy of 1-Pept/Dox NFs against colorectal carcinoma

HT29 tumor-bearing mice model was developed to study the antitumor effect in vivo of 1-Pept/Dox NFs. HT29 cell suspension (100 µL, 5.0 × 10^6^ cells) was mixed with an equal volume of Matrigel. Then, the mixtures were subcutaneously injected into the right back of male BALB/C nude mice (6–8 weeks, Jinan Pengyue Experimental Animal Breeding Co., Itd, China). As the tumor size approached 100 mm^3^, the mice were divided into four groups according to the random grouping principle: (1) PBS; (2) 1-Pept NFs; (3) Free Dox solutions; (4) 1-Pept/Dox NFs, respectively. 200 µL of the corresponding preparation was injected into each group by the peritumoral regions on days 1, 4, 7 and 10. The dosage was fixed as 13 µmol/kg for Dox moiety and 130 µmol/kg for 1-Pept. The tumor size and body weight of the mice were determined every three days. The tumor size was obtained using the equation V = L×W^2^/2, where L and W denote the length and width, respectively. At the end of the day 21st, the tumor tissues were collected for HE staining to observe morphological changes in tumor cells. The actin and tubulin staining of the cell sections was also conducted to study the cytoskeleton changes after drug treatment. The Ki-67 staining was conducted to observe the proliferative status of tumor cells. Meanwhile, the signals of TUNEL and expressions of activated caspase-3 were also measured by immunohistochemical staining.

### Biocompatibility assessment of 1-Pept/Dox NFs

To begin with, the biocompatibility of 1-Pept/Dox NFs was explored from hematological compatibility and histocompatibility. The hemolysis ratio of 1-Pept/Dox NFs was evaluated based on rabbit red blood cells (RBCs). The RBCs were incubated with 1-Pept/Dox NFs (1.0 wt%/0.1 eq) for 1 h. Then, the samples were first centrifuged to obtain the supernatant and measured the absorbance at 540 nm. Deionized water served as a positive group and 0.9% of the NaCl solution was utilized as a negative group. The hemolysis ratio of samples was obtained according to the hemolysis rate equation.

The clinical chemical parameters ALT and AST were detected to estimate the hepatotoxicity. The nephrotoxicity parameters of CREA and UA were also measured. About 200 µL 1-Pept/Dox NFs (3.0 wt%/0.3 eq) were injected at the right back of SD rats. The rat’s blood was drained after 7 days for centrifugation to obtain supernatants for further analysis.

The local tissue reactivity of 1-Pept/Dox NFs was evaluated. 200 µL of 1-Pept/Dox NFs was locally injected at the right back of the Balb/c mouse. The skin tissue at the injection site was collected after 7 days, and treated with hematoxylin eosin. The systemic toxicity test was observed in Balb/c mice. After 21 days of administration, the primary organs of the mice were collected and treated with hematoxylin eosin for further observation.

### Statistical analysis

All data differences were analyzed with one-way ANOVA. *P* < 0.05 is believed to be statistically significant.

## Conclusion

In summary, Tps-instructed nanoplatform 1-Pept/Dox NFs with cascading transformations were prepared by the co-assembly of Dox and a bioactive peptide 1-Pept for promoting chemotherapy against colon cancer. We first designed a Tps-responsive peptide derivative 1-Pept, consisting of the assembling domain, Tps cleavable domain, and Dox binding domain. The assembly behavior of 1-Pept changed significantly under the catalysis of Tps, including decreased CAC, MGC, formation of denser nanofibrillar networks, and improved rheological properties. To further explore its application in improving chemotherapeutic medication against colon cancer, Dox was selected as a model drug and co-assembled with 1-Pept into stable 1-Pept/Dox NFs *via* intermolecular electrostatic interactions. 1-Pept/Dox NFs exhibited enhanced assembly behaviors and excellent shear thinning performance, which makes it suitable as a drug depot. Additionally, 1-Pept/Dox NFs exhibited sustained drug release profiles in vitro, enhanced body retention, improved accumulation in tumor sites, and reduced distribution in normal organs compared with free Dox solution. Further results indicated that 1-Pept/Dox NFs exhibited intensified cellular uptake, which could be ascribed to caveolae-medicated endocytosis for avoiding lysosomal degradation and allowing perinuclear transportation of Dox, further enlarging the chemotherapeutic drug potency in the target areas, thus improving the anti-cancer efficacy. The released 1-Pept converted into Pept upon the intracellular overexpressed Tps catalysis and reassembled into denser Pept NFs, which triggered a cascade of effects including disruption of the cytoskeleton (actin and tubulin), mitochondrial damage, and activation of apoptosis-related proteins caspase-3. In the end, by the synergism of Pept NFs and Dox, caspase-3 was further activated, causing more significant damage to nuclear, thereby effectively killing cancer cells. Finally, the in vivo experiment showed that 1-Pept/Dox NFs could boost anti-tumor effects through multiple mechanisms and exhibited satisfactory biocompatibility. This research reported an enzyme-responsive nanoplatform with cascading transformations to promote chemotherapy against colon cancer.

This Tps-instructed bioactive peptide-based nanodrug has demonstrated great promise in colon cancer treatment by four unique features: (1) Efficient co-assembly of 1-Pept/Dox NFs marked with long-term retention in the body. (2) 1-Pept/Dox NFs exhibited an enhanced cellular uptake *via* caveolae-mediated endocytosis, which could avoid lysosomal degradation and further promote perinuclear transportation, thus retaining more drug potency in target areas. (3) In-situ control of the re-assembling process of 1-Pept with the aid of cell-derived Tps, facilitating the activating of a cascade of influences that lead to cell dysfunction. (4) The synergistic effects of bioactive peptides and chemotherapeutic drugs could promote tumor ablation. Considering specific enzymes expressed in the disease microenvironment, this approach introduced here could not only collaboratively combat colon cancer efficiently, but also provide a generalized strategy to improve the anticancer drug activity against other cancers, especially the drugs that act on the nucleus.

## Electronic supplementary material

Below is the link to the electronic supplementary material.

Supplementary Material 1

## Data Availability

No datasets were generated or analysed during the current study.
