# Supplementary material for: Trypsin-instructed bioactive peptide nanodrugs with cascading transformations to improve chemotherapy against colon cancer
Source: J Nanobiotechnology. 2025 Jan 31;23:66. doi: 10.1186/s12951-025-03143-1 (PMC11784115; doi:10.1186/s12951-025-03143-1)
Supplement: Supplementary file 1 — Supplementary Material 1 [file 12951_2025_3143_MOESM1_ESM.docx]

**Trypsin-Instructed Bioactive Peptide Nanodrugs with Cascading Transformations to Improve Chemotherapy against Colon Cancer**

Can Wu ^1,2^, XiaoWei Zhang ^2,3^, Manman Wang ^1^, Jinpan Sun ^1^, Jianfei Chen ^1^, Yanbin Guan ^1^, Xin Pang ^1*^

^1^ School of Pharmacy, Henan University of Chinese Medicine, Zhengzhou 450046, China.

^2^ Collaborative Innovation Center of Research and Development on the Whole Industry Chain of Yu-Yao， Henan Province, Henan University of Chinese Medicine, Zhengzhou 450046, China.

^3^ Academy of Chinese Medicine Science, Henan University of Chinese Medicine, Zhengzhou 450046, China.

**Corresponding Author**

*E-mail: pangxin116@163.com

**Table of Contents**

**Scheme S1** Structural elucidations of 1-Pept (A) and Pept (B).

**Fig. S1** TOF-MS of 1-Pept.

**Fig. S2** ^1^H-NMR spectrum of 1-Pept.

**Fig. S3** HPLC elution curves of 1-Pept, 1-Pept/Tps at 4 h and 1-Pept/Tps at 48 h.

**Fig. S4** MALDI-TOF-MS of 1-Pept/Tps solution at 48 h.

**Fig. S5** Circular dichroism spectra of 1-Pept and 1-Pept/Tps.

**Fig. S6** Dynamic strain scanning of 1-Pept NFs.

**Fig. S7** Measurement of intracellular Tps activity in different cells.

**Fig. S8** Optical image of 1-Pept solution after the addition of 1640 culture medium, NCM460 cell lysate, or HT29 cell lysate.

**Fig. S9** (A) TEM micrographs at the 12th hour of the collected HT29 cell lysates treated with 1-Pept. (B) TEM micrographs of collected HT29 cells pre-incubated with AEBSF for 2 h and then co-incubated with 1-Pept for 12 h. Bar, 1 μm.

**Fig. S10** (A) CLSM images of HT29 cells indicated mitochondrial membrane potential (ΔΨ_m_) with a JC-10 detection kit, which were treated by 1-Pept for 12 h or pre-incubated with AEBSF for 2 h and then co-incubated with 1-Pept for 12 h. Red, JC-10 aggregates, representing a high ΔΨ_m_; Green, JC-10 monomer, indicating a low ΔΨ_m_. Bar, 20 μm. (B) Fluorescence ratio of JC-10 red/JC-10 green of HT29 cells in Fig. S10A.

**Fig. S11** (A) CLSM images of intracellular caspase-3 expression in HT29 cells treated with 1-Pept for 12 h or pre-incubated with AEBSF for 2 h and then co-incubated with 1-Pept for 12 h. Green, GreenNuc™ caspase-3 substrate; Blue, Hoechst 33342-stained nucleus. Bar, 20 μm. (B) Relative fluorescence intensities of GreenNuc^TM^ caspase-3 substrate in 1-Pept/Dox NFs treated HT29 cells in Fig. S11A.

**Fig. S12** (A) Immunofluorescence staining of HT29 cells treated with 1-Pept for 12 h or pre-incubated with AEBSF for 2 h and then co-incubated with 1-Pept for 12 h for DNA damage tracking with Histone H2A.X. Green, Histone H2A.X; Blue, Hoechst 33342; Bar, 20 μm. (B) Relative fluorescence intensities of Aleax Fluor 488-conjugated Anti-Rabbit IgG(H+L) in 1-Pept/Dox NFs treated HT29 cells in Fig. S12A.

**Fig. S13** (A) TEM micrographs at the 12th hour of collected HT29 cell lysates treated with 1-Pept/Dox NFs. (B) TEM micrographs of collected HT29 cells pre-incubated with trypsin inhibitor (AEBSF) for 2 h and then co-incubated with 1-Pept/Dox NFs for 12 h. Bar, 1 μm.

**Fig. S14** CLSM images of HT29 cells treated with 1-Pept/Dox NFs for 1 h or 6 h. Red: 1-Pept/Dox NFs; Green: Lyso-Tracker Green; Bar, 20 μm.

**Fig. S15** Cell viability of SW620 (A), SW480 (B), T84 (C), and HCT-15 (D) cells after treatment with 1-Pept/Dox NFs or free Dox for 48 h.

**Fig. S16** CLSM images of intracellular actin fluorescence signals of HT29 cells treated with 1-Pept/Dox NFs or Dox solution for 12 h. Red, Alexa Fluor 633 phalloidin; Blue, DAPI; Bar, 20 μm.

**Fig. S17** Fluorescence immunoassay of tubulin in tumor tissues treated in different groups. Red, stained tubulin; Blue, DAPI for nuclei staining. Bar, 20 μm.

**Fig. S18** Percentages of caspase-3 positive cells in tumor tissue after treatment of various formulations.

**Fig. S19** Percentages of Tunel positive cells in tumor tissue after treatment of various formulations.

**Fig. S20** Percentages of Ki-67 positive cells in tumor tissue after treatment of various formulations.

**Table S1** The parameters of the 12-h Dox drug release from varying formulations by Ritger-Peppas modelling.

**Table S2** The parameters of 12-h 1-Pept drug release from varying formulations by Ritger-Peppas modelling.


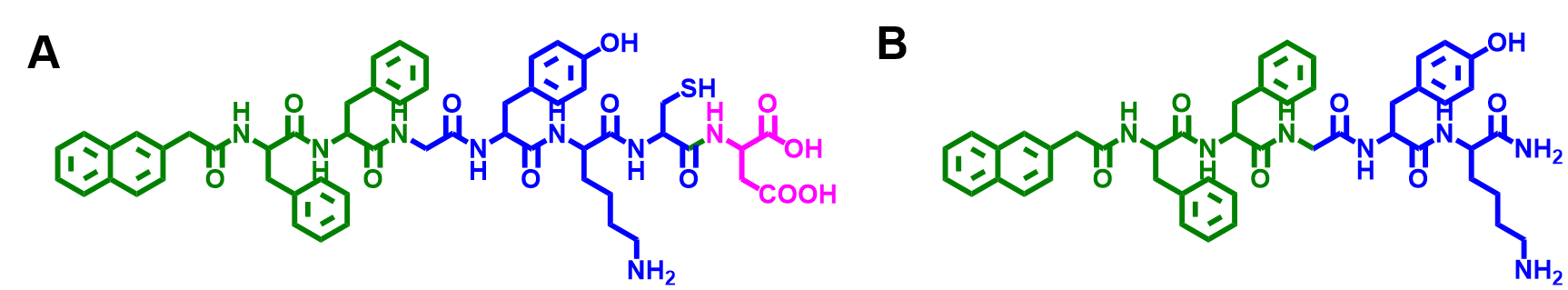


**Scheme S1** Structural elucidations of 1-Pept (A) and Pept (B).

**
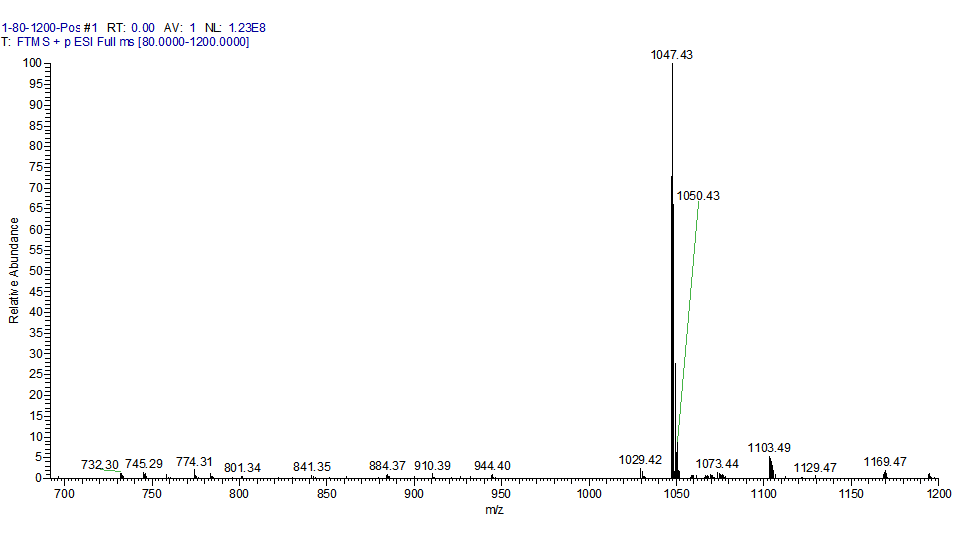
**

**Fig. S1** TOF-MS of 1-Pept. (ESI-MS: C_54_H_62_N_8_O_12_S, calc.MW =1046, obsvd. [M+H]^＋^= 1047.4, [M＋4H]^＋^=1050.4).


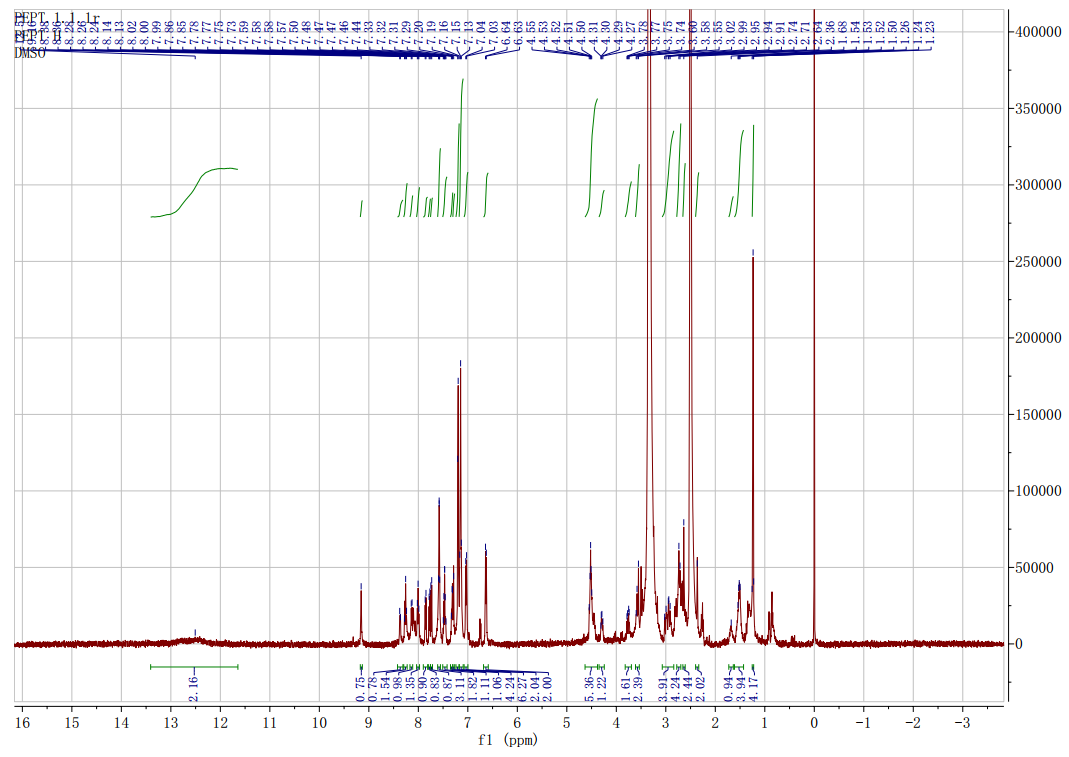


**Fig. S2** ^1^H-NMR spectrum of 1-Pept (300 MHz, ([D6] DMSO). C_54_H_62_N_8_O_12_S: 12.51 (S, 2 H), 9.16 (S, 1 H), 8.38-8.36 (d, 1 H, J = 6 HZ), 8.28-8.24 (m, 1 H), 8.14-8.13 (d, 1 H, J = 3 HZ), 8.02-7.99 (m, 1 H), 7.86-7.85 (d, 1 H, J = 3 HZ), 7.78-7.77 (d, 1 H, J = 3 HZ), 7.75-7.73 (d, 1 H, J = 6 HZ), 7.59-7.57 (m, 3 H), 7.50-7.44 (m, 2 H), 7.33-7.31 (m, 1 H), 7.29 (s, 1 H), 7.20-7.19 (d, 4 H, J = 3 HZ), 7.16-7.13 (m, 6 H), 7.04-7.03 (d, 2 H, J = 3 HZ), 6.64-6.63 (d, 2 H, J = 3 HZ), 4.55-4.50 (m, 5 H), 4.31-4.27 (m, 1 H), 3.78-3.74 (m, 2 H), 3.60-3.55 (m, 2 H), 3.02-2.91 (m, 4 H), 2.74-2.71 (d, 4 H, J = 9 HZ), 2.64 (s, 2 H), 2.36 (s, 2 H), 1.68 (s, 1 H), 1.54-1.50 (m, 4 H), 1.26-1.23 (m, 4 H).


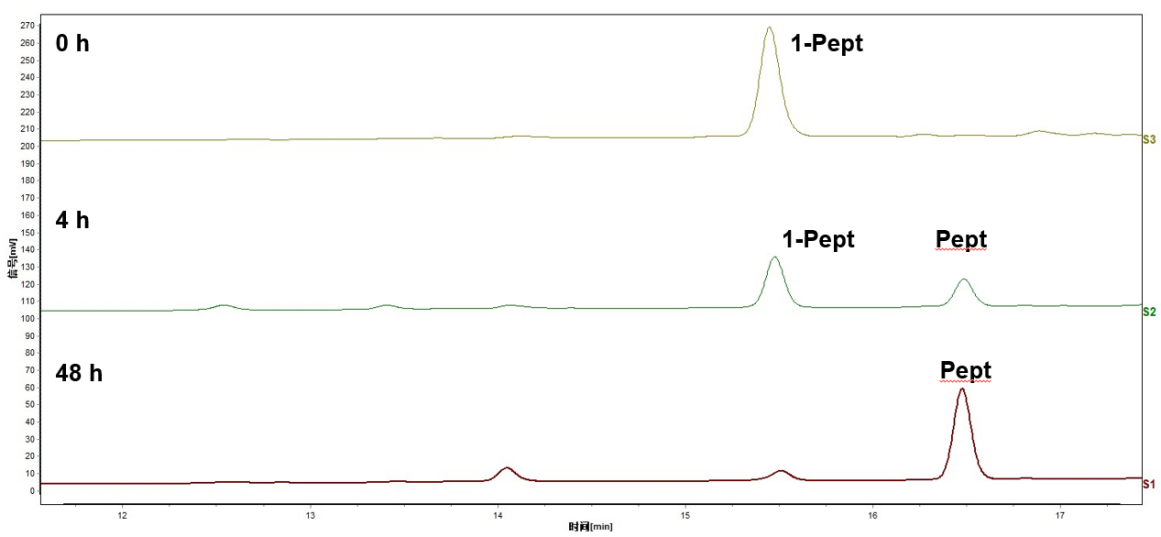


**Fig. S3** HPLC elution curves of 1-Pept, 1-Pept/Tps at 4 h and 1-Pept/Tps at 48 h.


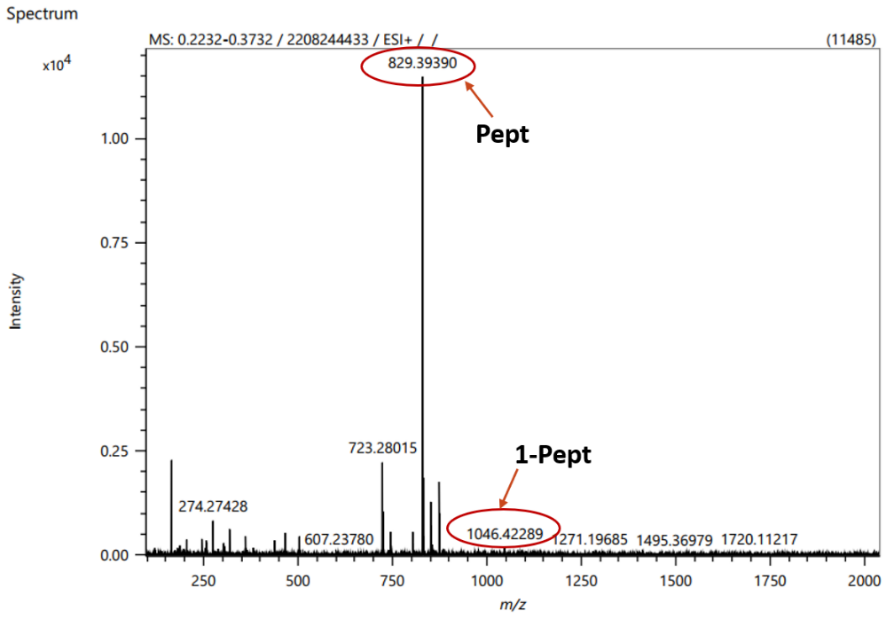


**Fig. S4** MALDI-TOF-MS of 1-Pept/Tps solution at 48 h, obsvd. [M]^＋^= 1046.4 for 1-Pept and [M＋2H]^＋^= 829.4 for Pept.


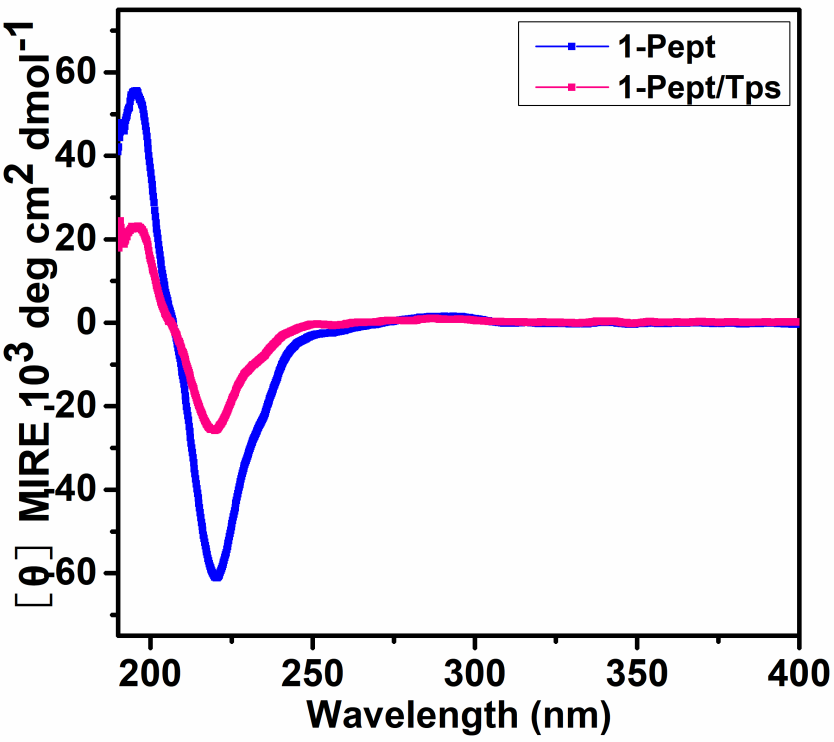


**Fig. S5** Circular dichroism spectra of 1-Pept and 1-Pept/Tps.

**
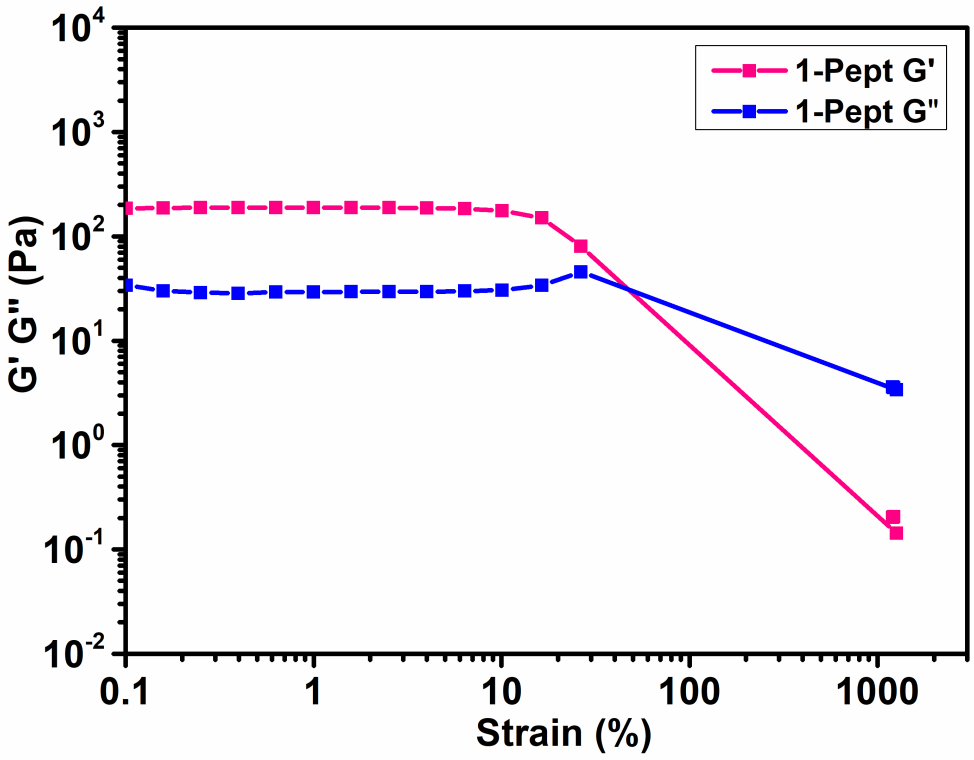
**

**Fig. S6** Dynamic strain scanning of 1-Pept NFs.


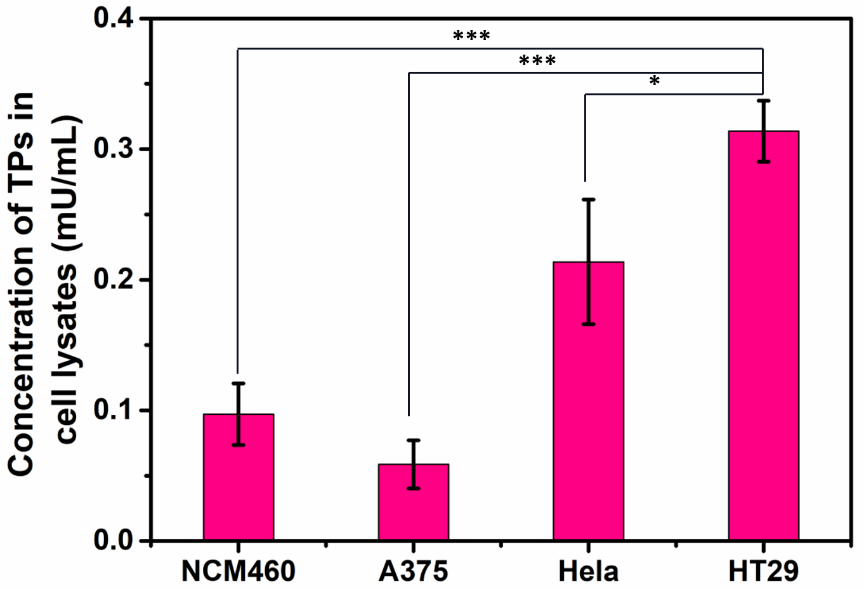


**Fig. S7** Measurement of intracellular Tps activity in different cells.


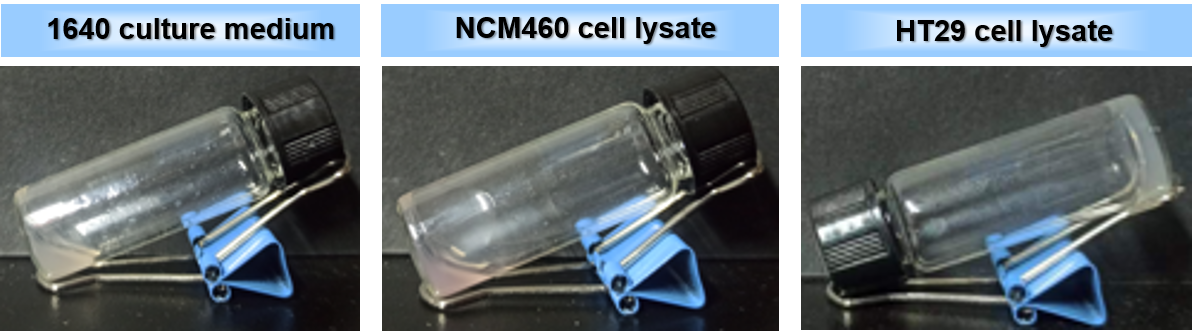


**Fig. S8** Optical image of 1-Pept solution after the addition of 1640 culture medium, NCM460 cell lysate, or HT29 cell lysate.


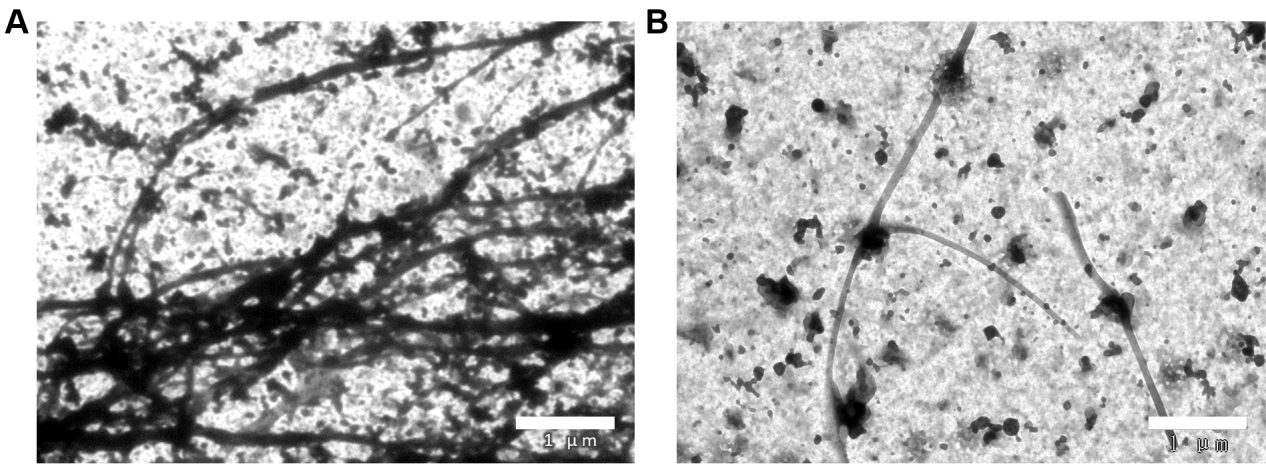


**Fig. S9** (A) TEM micrographs at the 12th hour of the collected HT29 cell lysates treated with 1-Pept. (B) TEM micrographs of collected HT29 cells pre-incubated with AEBSF for 2 h and then co-incubated with 1-Pept for 12 h. Bar, 1 μm.


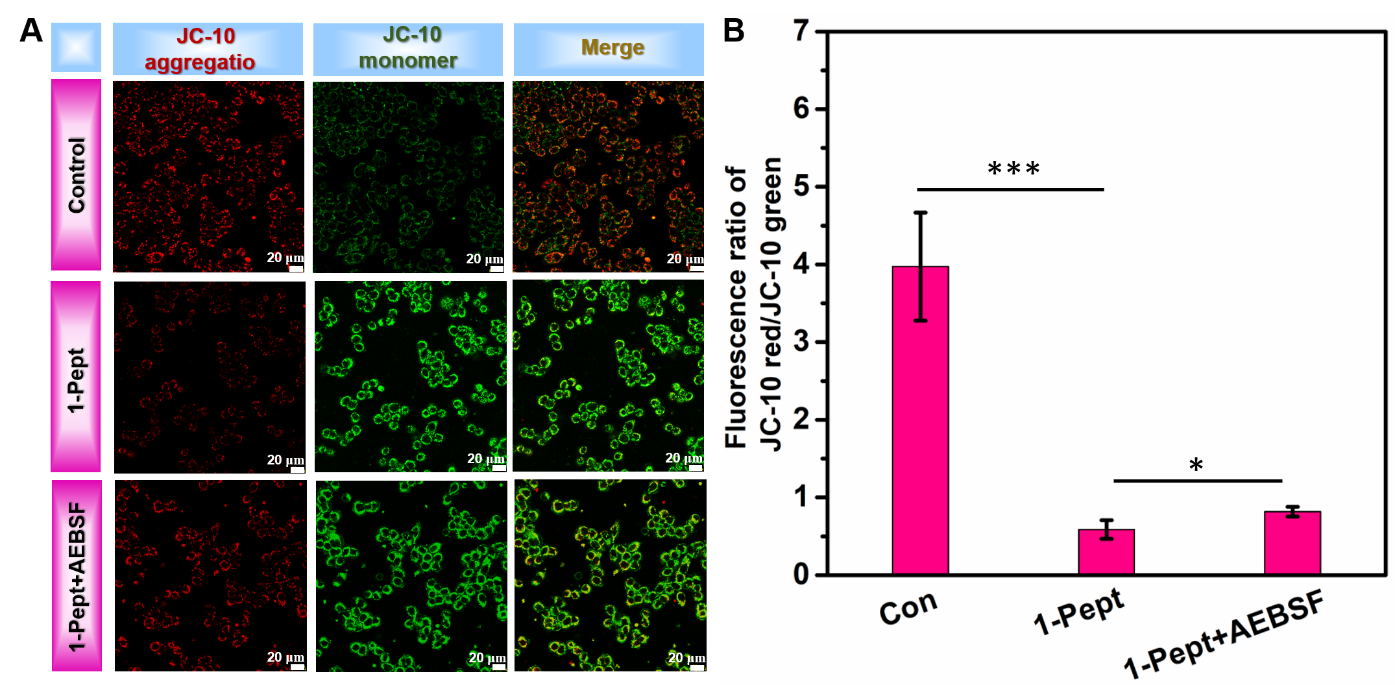


**Fig. S10** (A) CLSM images of HT29 cells indicated mitochondrial membrane potential (ΔΨ_m_) with a JC-10 detection kit, which were treated by 1-Pept for 12 h or pre-incubated with AEBSF for 2 h and then co-incubated with 1-Pept for 12 h. Red, JC-10 aggregates, representing a high ΔΨ_m_; Green, JC-10 monomer, indicating a low ΔΨ_m_. Bar, 20 μm. (B) Fluorescence ratio of JC-10 red/JC-10 green of HT29 cells in Fig. S10A.


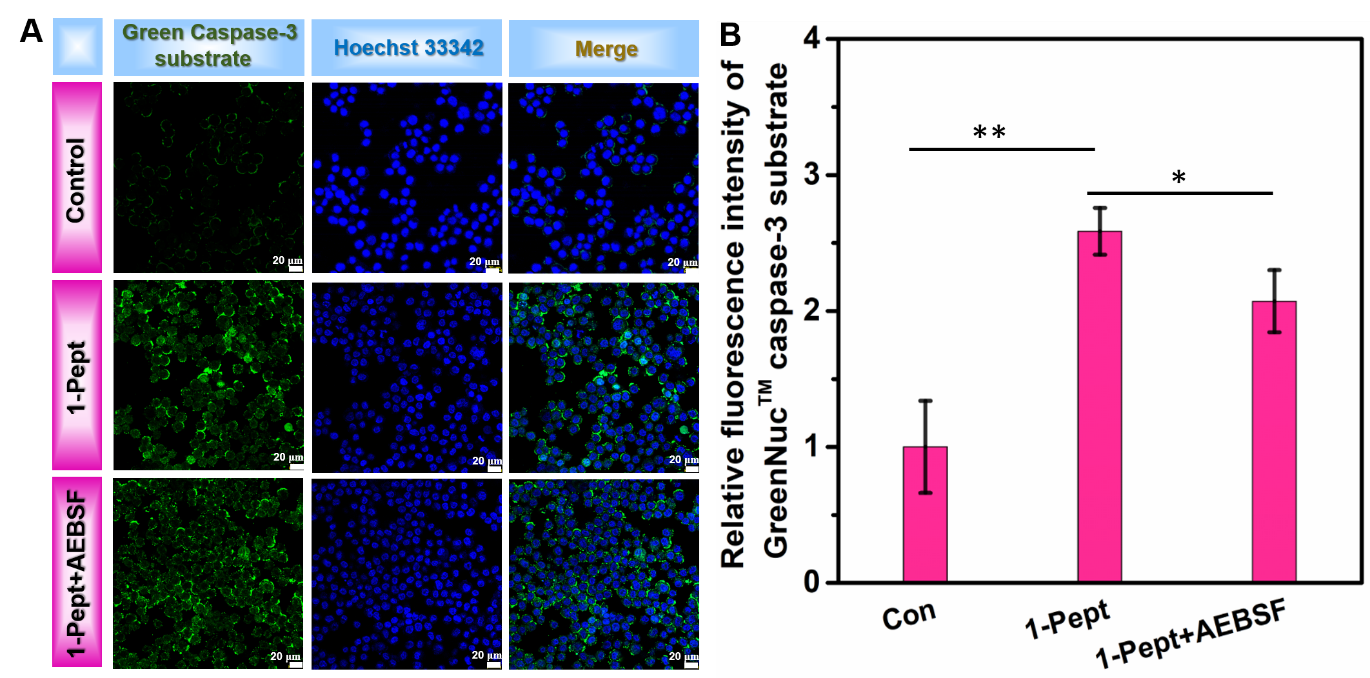


**Fig. S11** (A) CLSM images of intracellular caspase-3 expression in HT29 cells treated with 1-Pept for 12 h or pre-incubated with AEBSF for 2 h and then co-incubated with 1-Pept for 12 h. Green, GreenNuc™ caspase-3 substrate; Blue, Hoechst 33342-stained nucleus. Bar, 20 μm. (B) Relative fluorescence intensities of GreenNuc^TM^ caspase-3 substrate in 1-Pept/Dox NFs treated HT29 cells in Fig. S11A.


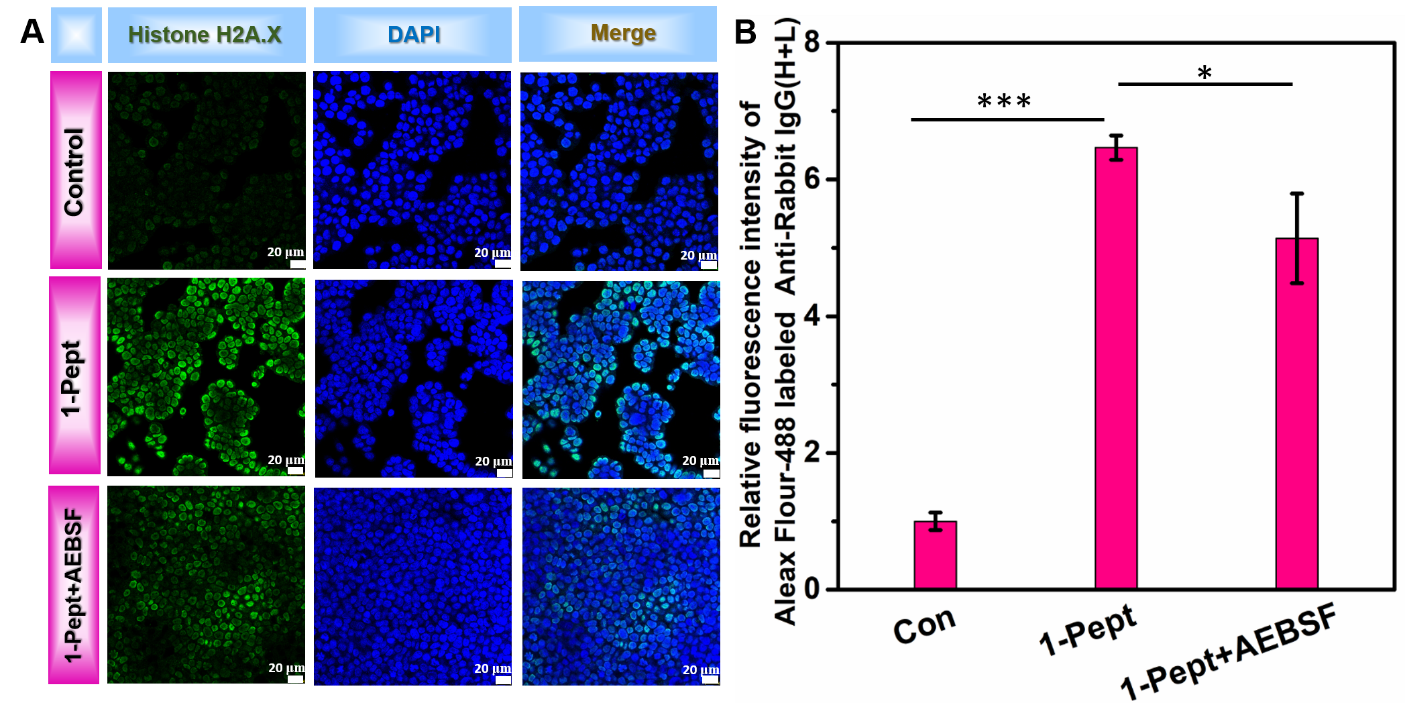


**Fig. S12** (A) Immunofluorescence staining of HT29 cells treated with 1-Pept for 12 h or pre-incubated with AEBSF for 2 h and then co-incubated with 1-Pept for 12 h for DNA damage tracking with Histone H2A.X. Green, Histone H2A.X; Blue, Hoechst 33342; Bar, 20 μm. (B) Relative fluorescence intensities of Aleax Fluor 488-conjugated Anti-Rabbit IgG(H+L) in 1-Pept/Dox NFs treated HT29 cells in Fig. S12A.


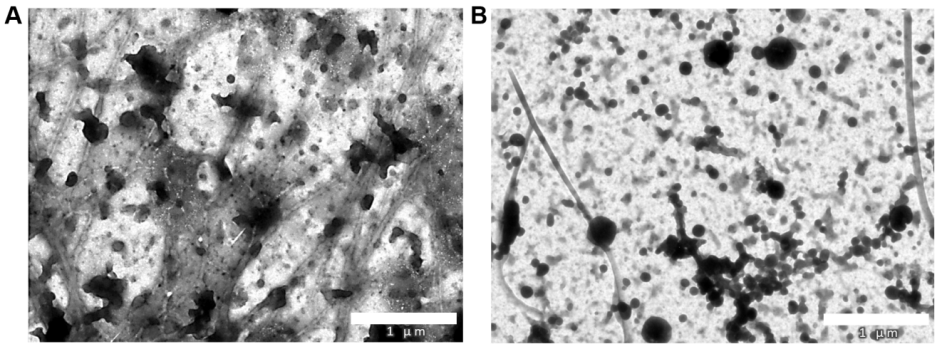


**Fig. S13** (A) TEM micrographs at the 12th hour of collected HT29 cell lysates treated with 1-Pept/Dox NFs. (B) TEM micrographs of collected HT29 cells pre-incubated with trypsin inhibitor (AEBSF) for 2 h and then co-incubated with 1-Pept/Dox NFs for 12 h. Bar, 1 μm.


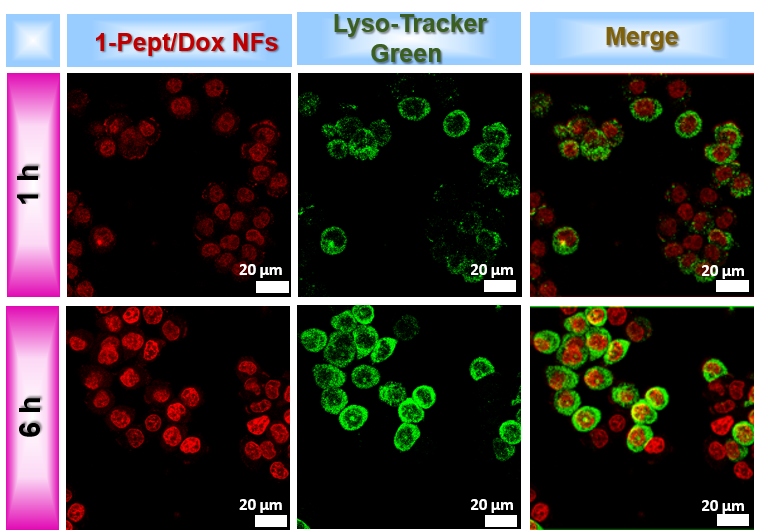


**Fig. S14** CLSM images of HT29 cells treated with 1-Pept/Dox NFs for 1 h or 6 h. Red: 1-Pept/Dox NFs; Green: Lyso-Tracker Green; Bar, 20 μm.

**
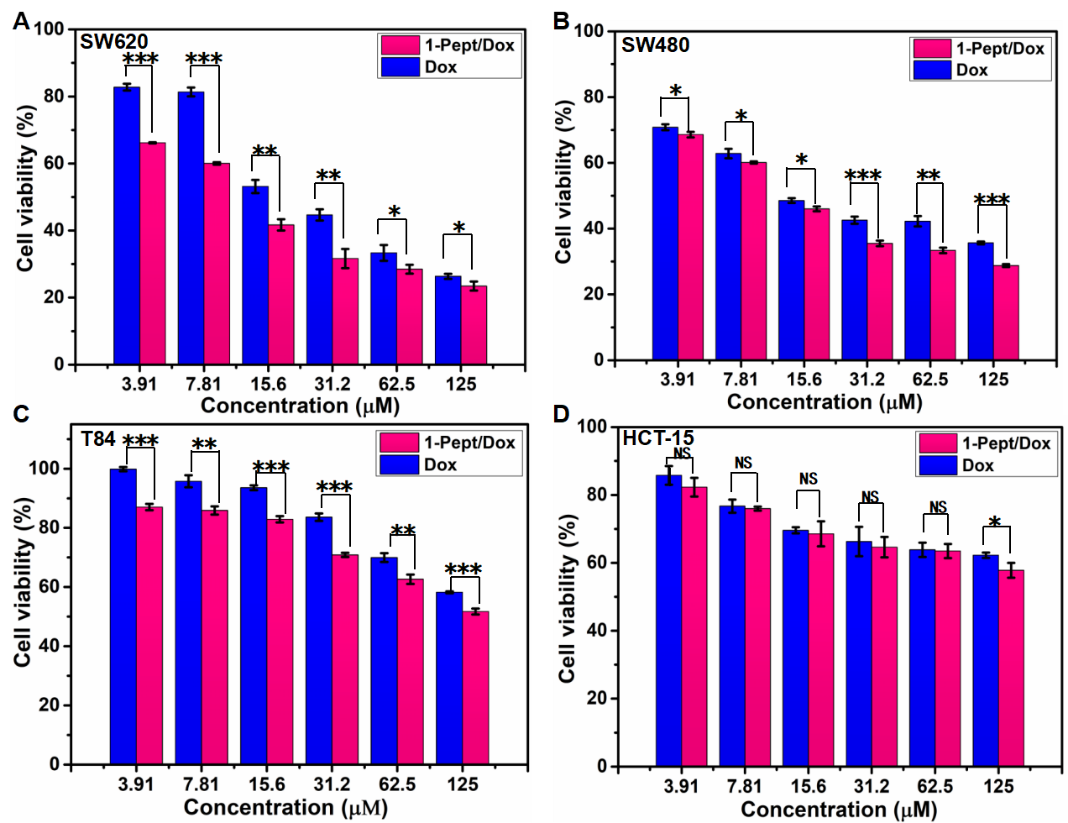
**

**Fig. S15** Cell viability of SW620 (A), SW480 (B), T84 (C), and HCT-15 (D) cells after treatment with 1-Pept/Dox NFs or free Dox for 48 h.


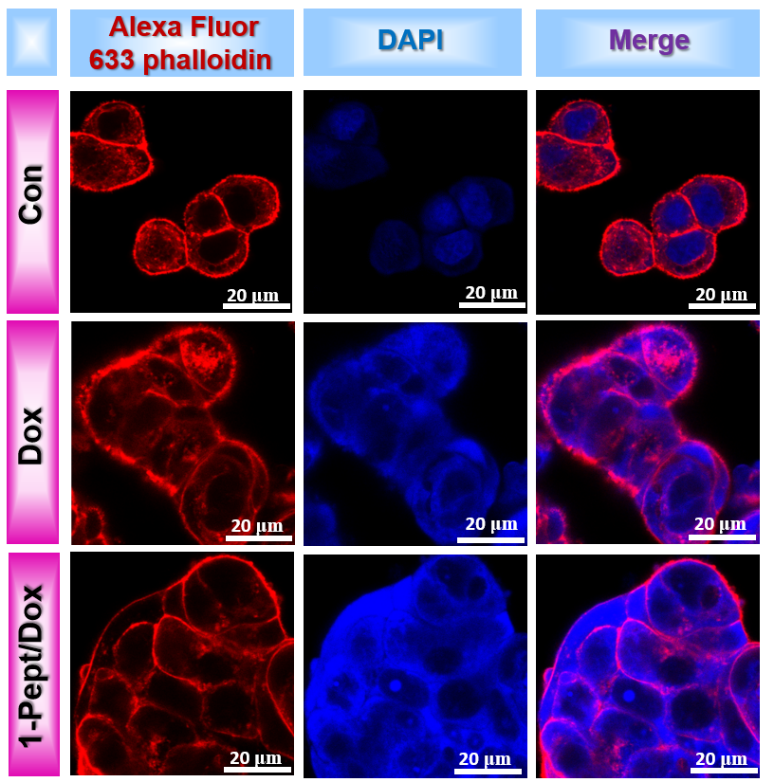


**Fig. S16** CLSM images of intracellular actin fluorescence signals of HT29 cells treated with 1-Pept/Dox NFs or Dox solution for 12 h. Red, Alexa Fluor 633 phalloidin; Blue, DAPI; Bar, 20 μm.


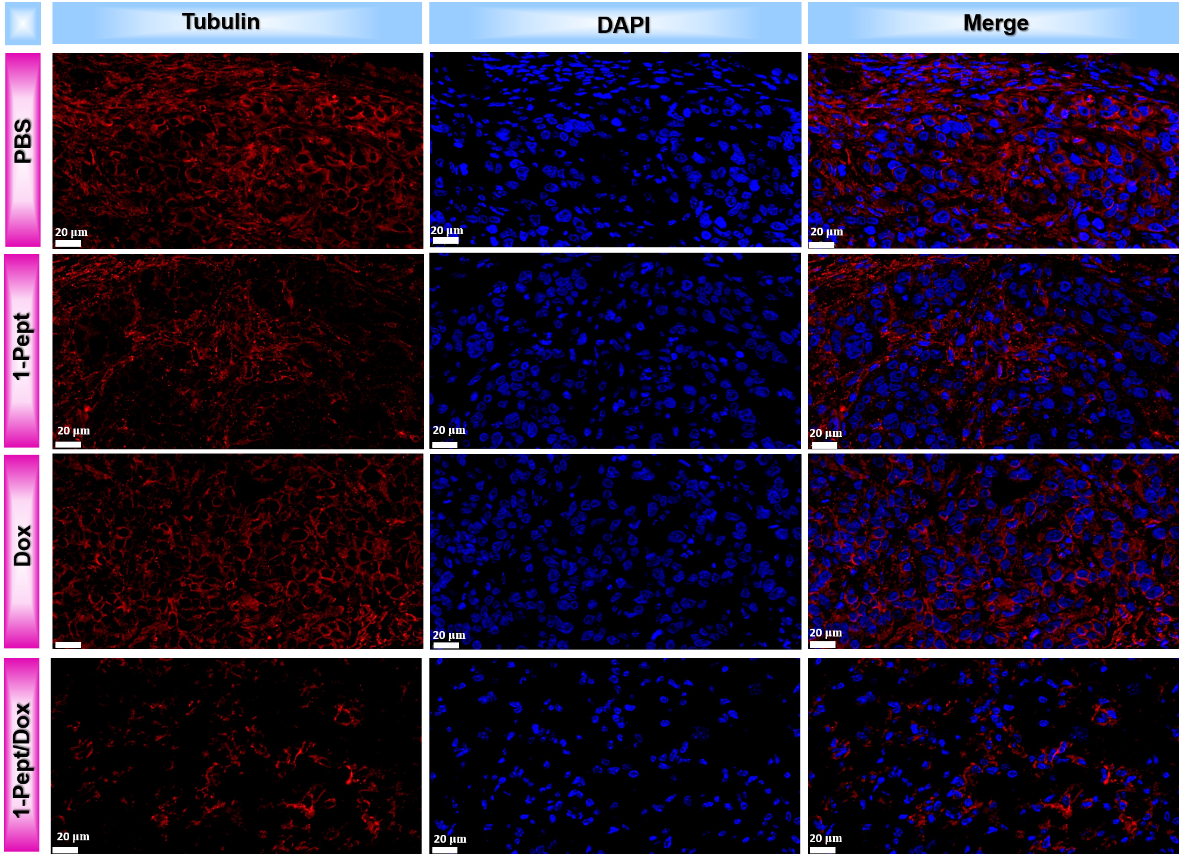


**Fig. S17** Fluorescence immunoassay of tubulin in tumor tissues treated in different groups. Red, stained tubulin; Blue, DAPI for nuclei staining. Bar, 20 μm.

**
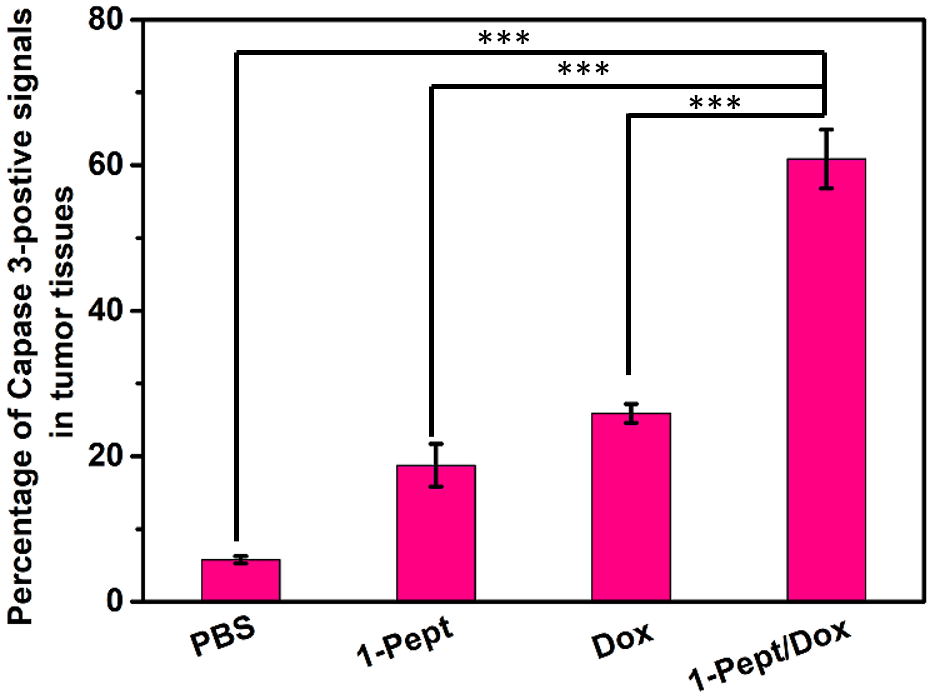
**

**Fig. S18** Percentages of caspase-3 positive cells in tumor tissue after treatment of various formulations.

**
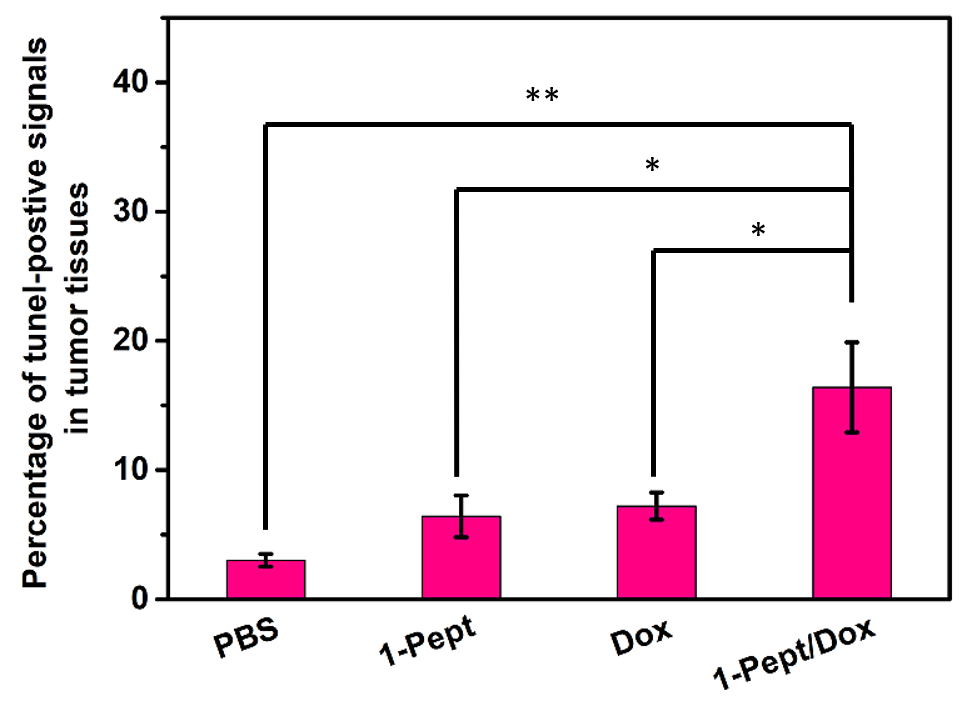
**

**Fig. S19** Percentages of Tunel positive cells in tumor tissue after treatment of various formulations.

**
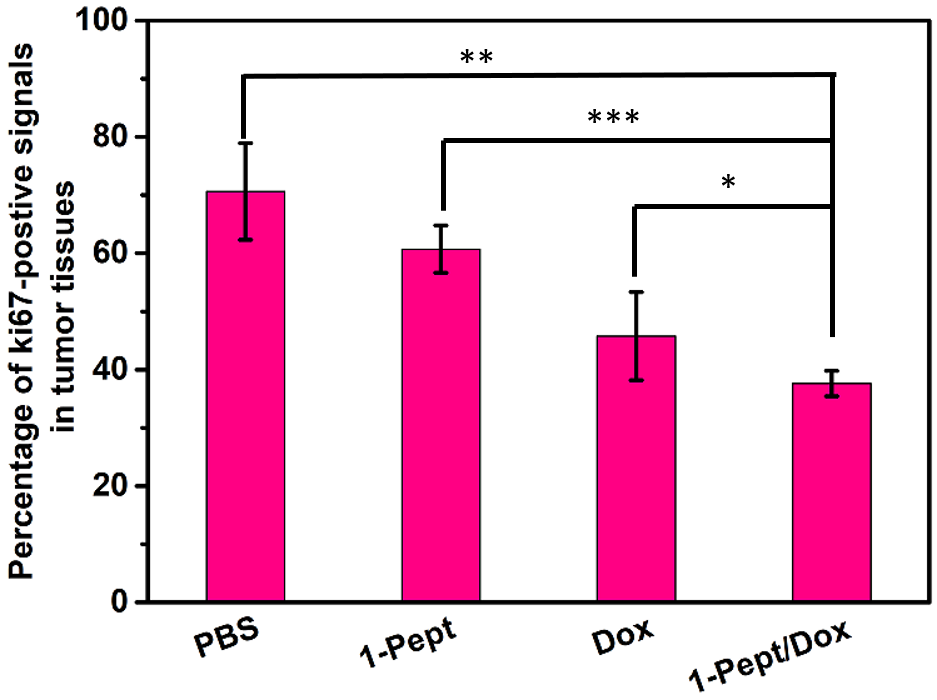
**

**Fig. S20** Percentages of Ki-67 positive cells in tumor tissue after treatment of various formulations.

**Table S1** The parameters of the 12-h Dox drug release from varying formulations by Ritger-Peppas modelling.

|  | n | k | R^2^ |
| --- | --- | --- | --- |
| Dox solution | 0.35 | 0.21 | 0.99 |
| 1-Pept/Dox NFs (pH 7.4) | 0.53 | 0.0069 | 0.98 |
| 1-Pept/Dox NFs (pH 6.5) | 0.54 | 0.013 | 0.97 |
| 1-Pept/Dox NFs (pH 5.5) | 0.58 | 0.016 | 0.98 |

**Table S2** The parameters of 12-h 1-Pept drug release from varying formulations by Ritger-Peppas modelling.

|  | n | k | R^2^ |
| --- | --- | --- | --- |
| 1-Pept/Dox NFs (pH 7.4) | 0.54 | 0.010 | 0.98 |
| 1-Pept/Dox NFs (pH 6.5) | 0.55 | 0.014 | 0.96 |
| 1-Pept/Dox NFs (pH 5.5) | 0.58 | 0.017 | 0.97 |
